# *PLXDC1* ^+^ Tumor‐Associated Pancreatic Stellate Cells Promote Desmoplastic and Immunosuppressive Niche in Pancreatic Ductal Adenocarcinoma

**DOI:** 10.1002/advs.202415756

**Published:** 2025-03-17

**Authors:** Yanhua Du, Yizhou Zhao, Judong Li, Jiaxin Wang, Shenglan You, Yao Zhang, Li Zhang, Jihong Yang, Hamid Alinejad‐Rokny, Shujie Cheng, Chenghao Shao, Duowu Zou, Youqiong Ye

**Affiliations:** ^1^ Center for Immune‐Related Diseases at Shanghai Institute of Immunology Department of Gastroenterology Ruijin Hospital Shanghai Jiao Tong University School of Medicine Shanghai 200001 China; ^2^ Shanghai Jiao Tong University School of Medicine‐Yale Institute for Immune Metabolism, State Key Laboratory of Systems Medicine for Cancer Shanghai Jiao Tong University School of Medicine Shanghai 20025 China; ^3^ Department of Pancreatic‐biliary Surgery Changzheng Hospital Naval Medical University Shanghai 200003 China; ^4^ Department of Hepatobiliary Surgery Hebei Key Laboratory of General Surgery for Digital Medicine Affiliated Hospital of Hebei University Baoding 071000 China; ^5^ UNSW BioMedical Machine Learning Lab (BML) School of Biomedical Engineering UNSW Sydney Sydney NSW 2052 Australia

**Keywords:** activation, heterogeneity, immunotherapy resistance, pancreatic stellate cells (PSCs); *PLXDC1* ^+^ tumor‐associated PSCs, single‐cell and spatial transcriptomes

## Abstract

Pancreatic stellate cells (PSCs) contribute to pancreatic ductal adenocarcinoma (PDAC) progression and therapeutic resistance, yet their detailed functions remain unclear. This study combined RNA sequencing and assay for transposase‐accessible chromatin using sequencing (ATAC‐seq) on sorted PSCs from adjacent normal and PDAC tissues to investigate their transcriptional and epigenetic activation. PSCs heterogeneity and functions are characterized through bulk, single‐cell, and spatial transcriptomes, as well as in situ sequencing. The clinical relevance of PSCs in immunotherapy is assessed using an in‐house immune‐checkpoint blockade (ICB) treatment cohort. Findings showed that stress and hypoxia signaling activated PSCs in PDAC. Three common PSCs (CPSCs) and four tumor‐associated PSCs (TPSCs) are identified, each with distinct functions. CPSCs differentiated into *CCL19* ^+^ TPSCs in immune‐enriched regions, *MYH11* ^+^ TPSCs in the stromal region, and *PLXDC1* ^+^ TPSCs, which exhibited cancer‐associated myofibroblasts (myCAFs) phenotype linked to poor prognosis. Notably, *PLXDC1* ^+^ TPSCs, located near aggressive *LRRC15* ^+^ myCAFs and *SPP1* ^+^ macrophages, formed a desmoplastic and immunosuppressive niche around the tumor boundary, promoting CD8 T cell exhaustion. Single‐cell transcriptomics of PDAC patients treated with ICB revealed that *PLXDC1* ^+^ TPSCs correlated with poor immunotherapy efficacy. Overall, this study provides key insights into PSCs in PDAC and potential therapeutic targets.

## Graphical abstract

This study uncovers the activation, heterogeneity, and regulatory roles of pancreatic stellate cells (PSCs) at single‐cell and spatial levels. It further identifies *PLXDC1* ^+^ PSCs near aggressive *LRRC15* ^+^ cancer‐associated myofibroblasts and *SPP1* ^+^ macrophages, forming a desmoplastic and immunosuppressive tumor niche that promotes *CD8* ^+^ T cell exhaustion, contributing to poor immunotherapy outcomes in pancreatic cancer.

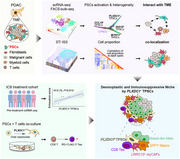

## 1 Introduction

Pancreatic ductal adenocarcinoma (PDAC) is highly lethal, accounting for over 90% of cases of pancreatic cancer.^[^ ^1^ ^]^ The stromal component, primarily composed of pancreatic stellate cells (PSCs) and cancer‐associated fibroblasts (CAFs), within the tumor microenvironment (TME) plays a crucial role in the development and treatment of PDAC.^[^ ^2^ ^]^ Quiescent PSCs play a crucial role in maintaining the normal physiological structure and exocrine function of the pancreas but become activated under pathological conditions, driven by factors such as TGF‐β and hypoxia, resulting in excessive collagen production and disruption of the extracellular matrix (ECM) balance.^[^ ^3^, ^4^ ^]^ While PSCs are conventionally regarded as the main source of CAFs in PDAC, recent findings from lineage‐tracing mouse model^[^ ^5^ ^]^ reveal that PSCs can only differentiate into a limited subset of *PDPN* ^+^ CAFs, suggesting that they may have CAF‐independent functions. Several studies have focused on the molecular and functional heterogeneity of pancreatic CAFs by single‐cell RNA sequencing (scRNA‐seq), such as the distinction between inflammatory (i) and myofibroblastic (my) CAFs.^[^ ^2^, ^6^ ^]^ However, within PDAC TME, our understanding of the regulation of PSC activation, the post‐activation status in vivo, and the transition to distinct phenotypes remains limited.

Studies reveal a symbiotic relationship between PSCs and cancer cells in the PDAC TME. PSCs can be activated and promote ECM production by cancer cell supernatants^[^ ^7^ ^]^ and activated PSCs further promote cancer cell proliferation.^[^ ^8^ ^]^ PSCs in PDAC are also involved in immunosuppression regulation by secreting CCL3 to recruit regulatory T cells (Tregs) and interleukin (IL)‐6 to promote STAT3‐dependent myeloid‐derived suppressor cell differentiation.^[^ ^9^ ^]^ Nevertheless, how PSCs interact with neighboring cell types to shape the PDAC TME, is currently unclear.

Considering PDAC fibrosis as a key factor in treatment resistance^[^ ^10^ ^]^ and the central role of PSCs in fibrosis, they are promising therapeutic targets. For example, targeting the VDR or RAR ligands can induce PSCs quiescence and enhance response to gemcitabine.^[^ ^11^, ^12^ ^]^ Prostaglandin E2 or L49H37 can suppress PSC proliferation and fibrotic stroma formation.^[^ ^13^, ^14^ ^]^ Activated PSCs regulate the suppressive immune microenvironment, suggesting that altering the functions of PSCs may restore the effectiveness of antitumor responses; however, the cross‐talk between PSCs and immune cells is very complex and requires further exploration. Most studies have focused on total PSCs, overlooking their heterogeneity. Thus, the complexity of PSCs in PDACs is still not fully characterized, hindering drug development targeting PSCs. Thus, a deeper understanding of PSCs heterogeneity and its across‐talk with TME will help develop attractive therapeutic strategies.

**Figure 1:**
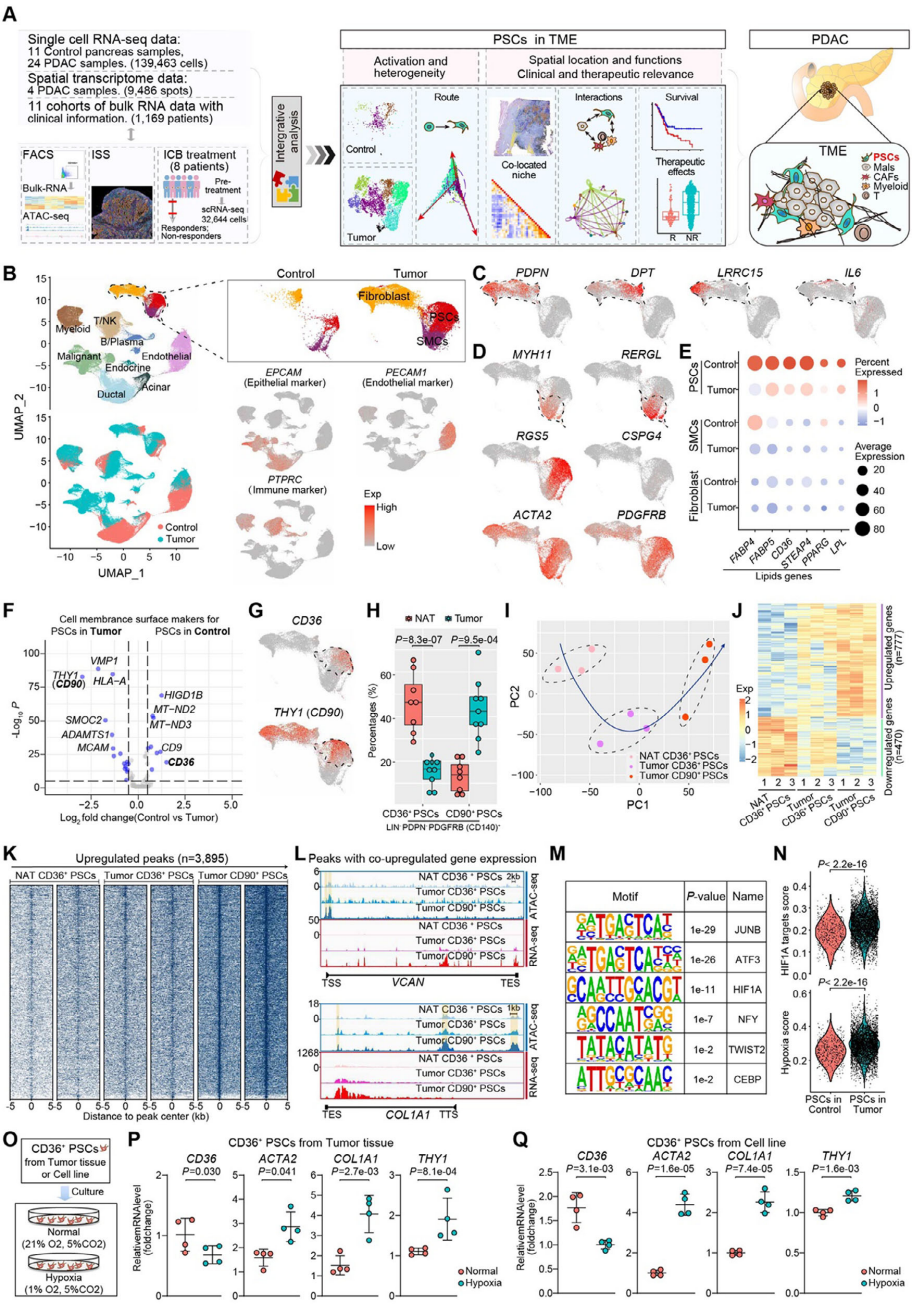
Transcriptomic and epigenetic reprogramming during the activation of PSCs. A) Diagrammatic overview of the study design. Single‐cell and spatial transcriptomic data were integrated and analyzed, correlated with clinical information in bulk transcriptomics from multiple cohorts, and incorporated with our in‐house FACS‐based bulk RNA‐seq and ATAC‐seq data, ISS data and scRNA data from ICB treatment cohort to resolve activation regulation and heterogeneity of PSCs and their spatial location, function, clinical and therapeutic relevance in the PDAC TME. B) UMAP plots of 139463 cells from 24 primary PDAC tumors and 11 control pancreases, showing 11 major cell types in different colors. *EPCAM1*, *PTPRC* (*CD45*), and *PECAM1* (*CD31*) are selectively expressed in epithelial cells, immune cells, and endothelial cells, respectively, distinct from mesenchymal stromal cell clusters (fibroblasts, PSCs, and SMCs). C, D) Feature plots showing the expression distribution of representative genes such as *PDPN*, *DPT*, *LRRC15*, and *IL6* for fibroblasts (C), and other characteristic genes for SMCs, PSCs, and pericytes (D) within mesenchymal stromal clusters. E) Dot plots showing the average expression (dot color) and percentage of cells expressed (dot size) of the lipid‐related genes for each mesenchymal stromal cluster from control pancreases and PDAC tumors. F) Volcano plot of single‐cell transcriptome data of PSCs derived from control pancreas and PDAC tumors. Only genes associated with the cell membrane surface were considered. Wilcoxon rank‐sum test was performed with Benjamini‐Hochberg–adjusted *P*‐values. G) Feature plots showing the expression distribution of *CD36* and *THY1* (*CD90*) within mesenchymal stromal clusters. H) The proportion of LIN^−^ PDPN^−^ PDGFRB (CD140)^+^ CD90^+^ PSCs and LIN^−^ PDPN^−^ PDGFRB (CD140)^+^ CD36^+^ PSCs in adjacent normal tissues (*n* = 8) and PDAC tumors (*n* = 9). Box middle lines, median; box limits, upper and lower quartiles; box whiskers, 1.53 the interquartile range. Statistical differences were determined using the Wilcoxon rank‐sum test. I) PCA of RNA‐seq data. Samples were analyzed in triplicate. J) Heatmap showing 777 upregulated and 470 downregulated genes during the activation of PSCs, which were defined as differentially expressed genes (DEGs) between CD36^+^ PSCs in adjacent normal tissues and CD90^+^ PSCs in PDAC tumors. K) Heatmap of ATAC‐seq tag densities were within ‐5/+5 kb genomic regions surrounding the centers of 3895 upregulated peaks during the activation of PSCs. Samples were prepared in duplicate. L) IGV view showing RNA‐seq and ATAC‐seq signaling profiles of the loci of upregulated genes (*COL1A1* and *VCAN* as examples). Shaded boxes represent upregulated peaks during the activation of PSCs. M) Transcription factor motif analysis of upregulated peaks on upregulated genes (peaks with co‐upregulated gene expression, *n* = 996) showing significantly enriched binding elements during the activation of PSCs. *p*‐values were calculated using hypergeometric distribution. N) Violin plots showing the distribution of average expression scores for HIF1A‐targeted genes and hypoxia‐associated genes in PSCs derived from control pancreas and PDAC tumors. Statistical differences were determined using the Wilcoxon rank‐sum test. (Table S6, Supporting Information). O) The culture of *CD36* ^+^ PSCs sorted from tumor tissues in PDAC patients or the *CD36* ^+^ PSCs cell line under hypoxic or normoxic conditions. P, Q) Dot plot with error bars showing relative mRNA expression changes of activation or quiescence marker genes by qPCR, comparing *CD36* ^+^ PSCs sorted from tumor patients (P) or a cell line (Q) cultured under normal versus hypoxia conditions. The error bars indicate the mean ± SD. Statistical difference was determined by a two‐tailed unpaired Student's t‐test.

When cultured in vitro, the characteristics of PSCs change, limiting the study of their heterogeneity and function in PDAC. Advances in single‐cell and spatial transcriptomic technologies offer opportunities to characterize PSCs in the TME. In this study (**Figure** **1**A), we integrated bulk, single‐cell, and transposase‐accessible chromatin using sequencing (ATAC‐seq) to reveal the transcriptional regulation and epigenetic dynamics of PSC activation and phenotypic transition, the heterogeneity of PSCs which included three common PSCs (CPSCs) and four tumor‐associated PSCs (TPSCs). Next, combined with spatial transcriptomics and in situ sequencing (ISS), distinct spatial distributions of PSC subclusters were identified, which interacted with cell types in TME to generate different niches and interactions. Importantly, we found that *PLXDC1* ^+^ TPSCs promoted the formation of a desmoplastic and immunosuppressive niche. Moreover, we determined that the *PLXDC1* ^+^ TPSCs were associated with immunotherapy efficacy by our in‐house immune‐checkpoint blockade (ICB) treatment cohort. Together, these results indicate the single‐cell and spatial heterogeneity of PSCs, highlight potential intercellular signals that control the localization and status of relevant cell types and guide the development of therapeutic strategies that target PSCs.

## 2 Results

### 2.1 Transcriptional and Epigenetic Activation of PSCs During PDAC Tumorigenesis

To characterize PSCs in the PDAC TME, we analyzed gene expression profiles of 139463 cells from 24 primary PDAC tumors and 11 normal pancreas samples (control), clustering them into 11 major types (Figure 1B–E; Figure S1A and Table S1, Supporting Information); see Experimental Section). Mesenchymal stromal cells were clearly distinguished form epithelial cells, immune cells, and endothelial cells (Figure 1B). *PNPN*, is commonly used for FACS of PDAC fibroblasts,^[^ ^6^ ^]^ is exclusively expressed in fibroblasts. Similarly, *DPT*, *LRRC15*, and *IL6*, mark progenitor fibroblasts, myCAF, and iCAFs respectively,^[^ ^2^, ^15^ ^]^ show corresponding expression patterns in fibroblasts but not in other stromal cell types (Figure 1C). *PDGFRB* and *RSG5* are markers for hepatic and pancreatic stellate cells and are also expressed in smooth muscle cells (SMCs) and pericytes.^[^ ^16^, ^17^, ^18^ ^]^ Due to the absence of clear PSCs markers, we were compelled to analyze markers of other known stromal cell types. Our analysis showed no enrichment for pericyte marker *CSPG4* and SMCs markers *MYH11* and *RERGL* (Figure 1D). Furthermore, we found significant enrichment of lipid‐related genes (e.g., *FABP4*, *FABP5*, *CD36*) in PSCs from control pancreases, consistent with the metabolic profile of quiescent PSCs (Figure 1E). Notably, Sherman et al. used a *Fabp4*‐Cre mouse model to trace the fate of PSCs.^[^ ^5^ ^]^ In addition, *ACTA2* as an activated PSCs marker exhibits stronger expression in the PDAC samples (Figure 1D). Therefore, we've defined clusters of PSCs at the single‐cell level, distinct from *PDPN*‐positive fibroblasts.

In PDAC tumor tissues, the proportion of PSCs was higher and the enrichment score of PSC activation signatures^[^ ^11^ ^]^ was heightened (Figures S1B,C, Supporting Information). To reveal the regulatory mechanisms, we compared the expression profiles of PSCs in control and PDAC tissues. We found that cell‐membrane surface CD36 was enriched in the control pancreas, while THY1 (CD90) was enriched in PDAC tissues (Figure 1F), indicating the quiescent and activated states of PSCs,^[^ ^11^ ^]^ respectively. LIN^−^ PDPN^−^ PDGFRB (CD140)^+^ CD36^+^ PSCs and LIN^−^ PDPN^−^ PDGFRB (CD140)^+^ CD90^+^ PSCs were isolated to validate that activated PSCs (CD90^+^ PSCs) were enriched in PDAC tissues, while quiescent PSCs (CD36^+^ PSCs) were enriched in adjacent normal tissues (NATs) (Figure 1G,H; Figure S1D and Table S2, Supporting Information). Next, the heterogeneous states of PSCs were characterized by their transcriptional and chromatin‐accessibility landscape. Principal component analysis (PCA) clearly demonstrated an activated transition from CD36^+^ PSCs in NAT to CD36^+^ PSCs in PDAC tissues and then to CD90^+^ PSCs (Figure 1I). Differential expressed genes (DEGs) analysis during PSC activation revealed 777 upregulated genes associated with processes such as ECM formation, cell adhesion, inflammatory responses, and MAPK signaling, while 470 downregulated genes were linked to homeostasis, lipid storage, and vascular process (Figures 1J; Figure S1E and Table S3, Supporting Information). Then, the signature scores based on the upregulated genes in activated PSCs were confirmed to be higher in tumor tissues, while the downregulated genes were enriched in control tissues (Figure S1F, Supporting Information).

Further, we profiled the open chromatin regions (OCRs) during PSC activation based on ATAC‐seq, and 42551, 33778, and 25847 OCRs were identified in NAT CD36^+^ PSCs, PDAC CD36^+^ PSCs, and PDAC CD90^+^ PSCs, respectively (Figure S1G, Supporting Information). Furthermore, 12211 upregulated and 3895 downregulated OCRs were found in activated PSCs (Figure 1K; Figure S1H and Table S4, Supporting Information). These DE OCRs mainly existed in the promoter, exon, and 5′‐UTR (FigureS1I, Supporting Information), suggesting their regulatory roles. Through the integration of DE OCRs and DEGs, 996 peaks associated with co‐upregulated gene expression and 835 peaks associated with co‐downregulated gene expression were ultimately identified (Figure 1L; Figure S1G–J, Supporting Information; see Experimental Section). For example, *HES1* and *VIM* associated with quiescent PSCs showed reduced chromatin accessibility downstream and upstream of their transcription start site and reduced gene expression, whereas *VCAN* and *COL1A1* associated with activated PSCs showed an opposite pattern. To identify key regulatory factors during PSC activation, motif analysis on OCRs was conducted, where gene expression and DNA accessibility were synchronously altered. The results indicated that the synchronously upregulated regions were significantly enriched with hypoxia‐related factors (HIF1A, TWIST2, etc.) and stress response factors (FOS, ATF3, etc.), whereas the synchronously downregulated regions were notably enriched with regulatory factors including STAT3 and VDR (Figure 1M; Figure S1K, Supporting Information). Furthermore, we scored the genes targeted by HIF1A elements as well as hypoxia‐related genes and found a significant enrichment in PSCs from PDAC tumors (Figure 1N). These findings highlighted the prominent regulatory roles of stress and hypoxia signaling in promoting PSC activation. To investigate whether hypoxic conditions promote the activation of quiescent PSCs, we sorted CD36^+^ PSCs from PDAC patients or obtained them from a CD36^+^ PSC cell line and cultured them under normoxic or hypoxic conditions (Figure 1O; see Experimental Section). We then measured the expression of markers of activation and quiescence by qPCR. The results showed significant upregulation of activation markers (*ACTA2, COL1A1, and THY1*) and downregulation of the quiescence marker CD36 (Figure 1P,Q), confirming that hypoxia activates PSCs.

### 2.2 Single‐Cell Transcriptomic Profiling Reveals Heterogeneity of PSCs in PDAC

**Figure 2:**
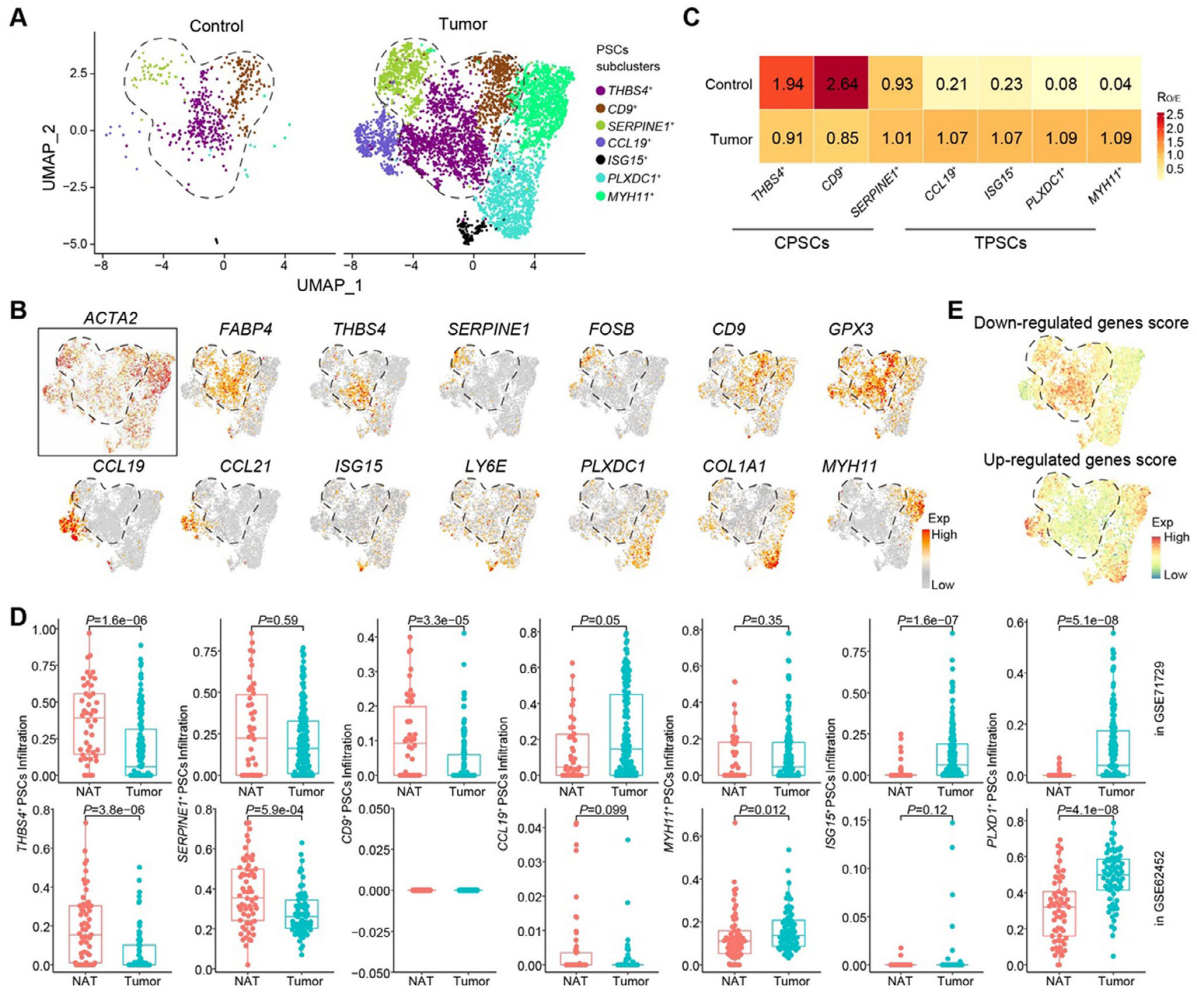
Characterization of common and tumor‐associated PSCs in control pancreas and PDAC samples using single‐cell transcriptome. A) Uniform Manifold Approximation and Projection (UMAP) plots showing 7549 PSCs from human control pancreas and PDAC tumors are clustered into seven subclusters. Each dot represents one cell. Cells are color‐coded by cell clusters accordingly. B) Feature plots showing the expression distribution of representative genes in each PSC subcluster. C) Tissue prevalence of each PSC subcluster estimated based on the R_O/E_ scores. CPSCs, common pancreatic stellate cells; TPSCs, tumor‐associated pancreatic stellate cells. D) Box plot comparing the adjacent normal tissue (N) and tumor (T) abundance of each PSC subcluster in the GSE71729 (*n* = 46 for N, *n* = 145 for T) and GSE62452 (*n* = 61 for N, *n* = 69 for T) cohorts and predicted using CIBERSORTx. ^[^ ^21^ ^]^ Statistical differences were determined using the Wilcoxon rank‐sum test. E) Feature plots showing the distribution of average expression score for upregulated and downregulated genes defined in Figure 1J in each PSC subcluster.

To explore the heterogeneity of PSCs in PDAC, we extracted 7549 PSCs, corrected for batch effects using Harmony^[^ ^19^ ^]^ and RPCA^[^ ^20^ ^]^ to ensure consistency (Figure S2A–F, Supporting Information), and constructed a PSCs atlas. The PSCs were then categorized into seven subclusters, including *THBS4^+^* PSCs (*THBS4*, *COLEC11*, *STEAP4*); *SERPINE1^+^* PSCs (*SERPINE1*, *CCL2*, *CTGF*); *CD9^+^* PSCs (*CD9*, *RASD1*, *RGCC*); *CCL19^+^* PSCs (*CCL19*, *CCL21*, *MFAP4*); *ISG15^+^* PSCs (*ISG15*, *IFIF2*, *IFIT3*); *PLXDC1^+^* PSCs (*PLXDC1*, *COL1A1*, *FN1*); and *MYH11^+^* PSCs (*MYH11*, *S100A4*, *TAGLN*) (**Figure** **2**A,B; Figure S2G and Table S5, Supporting Information). Next, observed versus expected cell numbers within each cluster (R_O/E_ score) were used to compare different compositions of PSC subclusters (see Experimental Section). Four types of PSCs (*CCL19^+^*, *ISG15^+^*, *PLXDC1^+^*, and *MYH11^+^*) were identified as TPSCs with high R_O/E_ scores in tumor tissues, while remained three PSC subclusters were identified as CPSCs, mainly in the control pancreas and persist in tumor tissues (Figure 2C). This was validated by comparing the abundance of each PSC subcluster between adjacent normal and tumor tissues using the CIBERSORTx^[^ ^21^ ^]^ (Figure 2D).

Further, we observed the exclusive expression of *FABP4* in CPSCs, which serves as a reliable quiescent marker for tracking PSC fate^[^ ^5^ ^]^ (Figure 2B). Next, we assessed the enrichment of gene sets associated with CD36^+^ and CD90^+^ PSCs identified via RNA‐seq (Figure 1J). Notably, the CD36^+^ PSC‐related gene set, indicating quiescence, was significantly enriched in CPSCs, while the CD90+ PSC‐related gene set, reflecting activation, was significantly enriched in TPSCs (Figure 2E). Moreover, we observed significant enrichment of targets related to PSCs activation and hypoxia (Figure S2H, Supporting Information) in TPSCs, suggesting their distinct molecular features from CPSCs.

### 2.3 Three Distinct Subclusters of TPSCs with myCAF‐like, SMC‐like and Inflammation‐Associated Features

CPSCs typically remain quiescent in the normal pancreas. While the molecular and functional heterogeneity of these cells at the single‐cell level is limited, we identified and characterized three CPSCs subclusters in control pancreatic tissues (Figure 2A; Figure S3A,B, Supporting Information). Among these subclusters, *THBS4* ^+^ CPSCs demonstrated cellular plasticity and play roles in tissue homeostasis and stress response, whereas *CD9* ^+^ CPSCs primarily contribute to energy metabolism and muscle construct. Interestingly, *SERPINE1* ^+^ CPSCs express numerous cytokines and are associated with vascular development, resembling the phenotype of vascular PSCs.^[^ ^22^, ^23^ ^]^ Additionally, during the progression of PDAC, angiogenesis and cytokine secretion by *SERPINE1* ^+^ CPSCs were enhanced (Figure S3C, Supporting Information), indicating their potential involvement in remodeling to regulate tumorigenesis‐related pathways such as angiogenesis.

**Figure 3:**
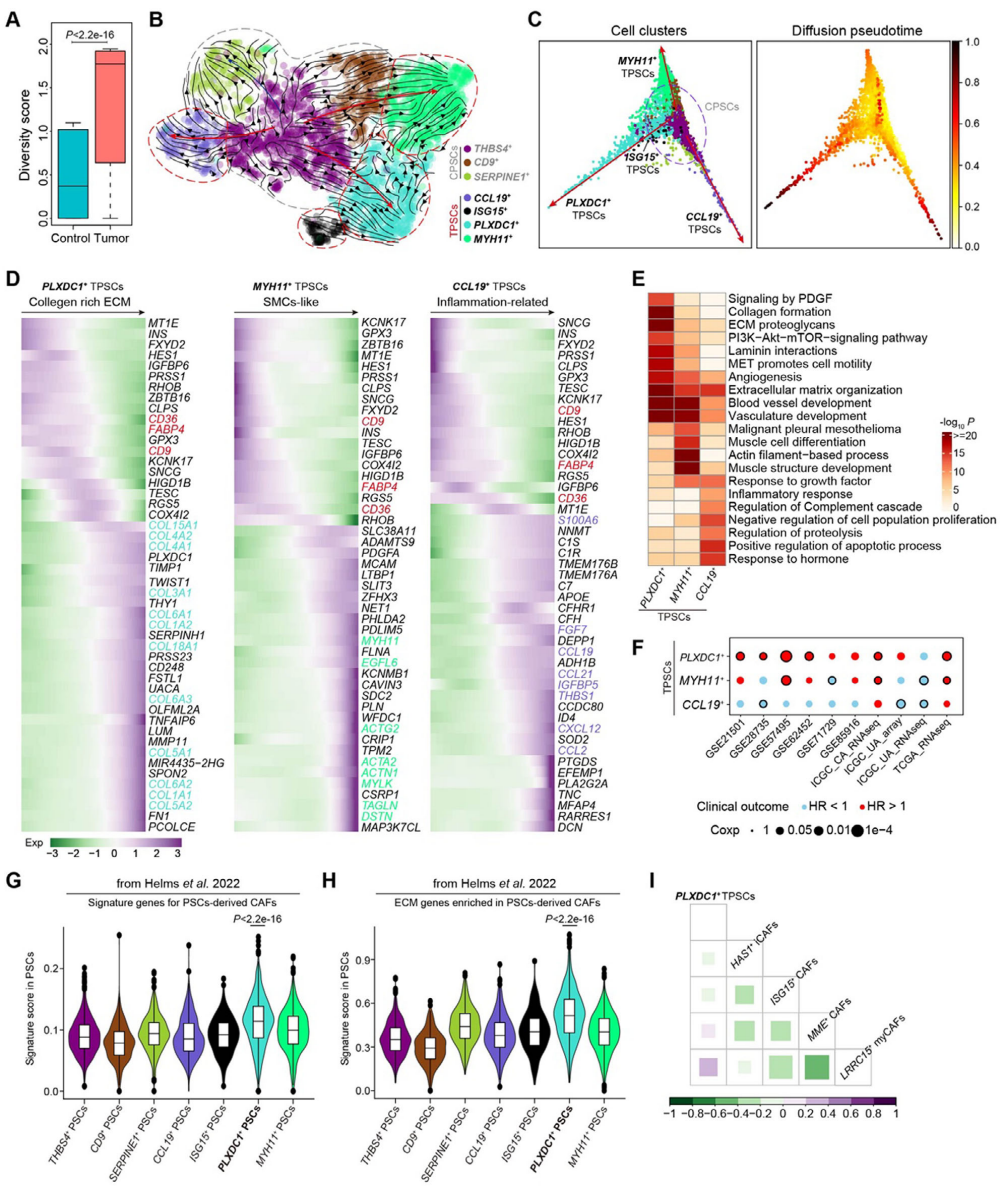
Three distinct subclusters of TPSCs with myCAF‐like, SMCs‐like, and inflammation‐associated features. A) Box plot comparison of gene expression diversity scores of PSCs from human control pancreas and PDAC tumors using the Shannon Diversity Index. Statistical differences were determined using the Wilcoxon rank‐sum test. B) UMAP visualization of subclusters of PSCs in PDAC samples using RNA velocity analysis based on standard splicing kinetics. Cells are color‐coded by subclusters. Streamlines represent integration paths connecting local projections from observed states to inferred future states. The thickness of the streamlines indicates the magnitude of the velocity. C) Diffusion map coordinates used to analyze subclusters of PSCs in PDAC samples, showing three different trajectories toward *PLXDC1* ^+^ TPSCs, *MYH11* ^+^ TPSCs, and *CCL19* ^+^ TPSCs (left panel). The right panel shows the pseudotime‐assigned color code of cells. D) Heatmap showing gene expression dynamics along the PSC lineage trajectories toward *PLXDC1* ^+^ TPSCs, *MYH11* ^+^ TPSCs, and *CCL19* ^+^ TPSCs. Representative genes from each cluster are shown, and the expression value for each gene is normalized to a Z‐score to facilitate the comparison of gene dynamics. E) Representative Gene Ontology (GO) terms enriched in different TPSC subclusters. Hypergeometric test was performed using Benjamini‐Hochberg–adjusted *p*‐values. F) Dot plot displaying the HR for overall survival (OS) of each TPSC subcluster in ten independent PDAC datasets. Dots with black circles indicate statistical significance. HR, hazard ratio; Coxp, *p*‐value calculated using Cox proportional hazards regression. G, H) Violin plots comparing specific gene set scores between *PLXDC1* ^+^ TPSCs and other TPSCs. The two gene sets used therein, including signature genes for PSC‐derived CAFs (G) and ECM genes enriched in PSC‐derived CAFs (H), were downloaded from Helms et al. ^[^ ^5^ ^]^ The scores were calculated as the average gene expression of each gene set. Statistical differences were determined using the Wilcoxon rank‐sum test. I) Dot plots showing Pearson's correlations between the abundance of *PLXDC1* ^+^ TPSCs and each CAF subcluster in 1169 PDAC samples predicted using CIBERSORTx.^[^ ^21^ ^]^ The size and color indicate the Pearson correlation coefficient.

PSCs undergo activation in PDAC and exhibit a greater diversity of gene expression compared to control counterparts (**Figure** **3**A), indicating high heterogeneity of PSCs within TME. Alongside *ISG15* ^+^ TPSCs, showing high expression of interferon‐stimulated genes possibly due to stress or stimulation, we defined three distinct subclusters of TPSCs. Utilizing RNA velocity analysis, we characterized their heterogeneity and plasticity, identifying three discrete differentiation paths from CPSCs to *PLXDC1* ^+^, *MYH11* ^+^, and *CCL19* ^+^ TPSCs (Figure 3B). The result was validated via diffusion map–based and PHATE‐based pseudo‐temporal analysis (Figure 3C; Figure S3D, Supporting Information). Subsequently, we extracted expression patterns along each trajectory path, revealing the activation of ECM‐related genes leading to *PLXDC1* ^+^ TPSCs, consistent with a classical myofibroblast phenotype; upregulation of muscle‐related genes towards *MYH11* ^+^ TPSCs, suggesting an SMC‐like phenotype; activation of multiple inflammatory and chemotactic factors on the path to *CCL19* ^+^ PSCs, indicating an inflammatory phenotype (Figure 3D,E). Furthermore, in nine independent PDAC cohorts comprising 1169 samples, we identified *PLXDC1* ^+^ TPSCs as unfavorable prognostic markers, with six cohorts showing statistical significance (Figure 3F; Figure S3E,F, Supporting Information), implying their potential involvement in tumor progression.

To assess the myofibroblast characteristics of *PLXDC1* ^+^ TPSCs further, we obtained signature genes and ECM genes enriched in PSC‐derived CAFs^[^ ^5^ ^]^ and calculated their expression score in PSCs. We found *PLXDC1* ^+^ TPSCs exhibited significantly higher scores for PSC‐derived CAF characteristics and ECM features compared to other PSC subclusters (Figure 3G,H), suggesting their potential transition into CAFs. Then, we classified fibroblasts into six subclusters: *PI16* ^+^ and *DPT* ^+^ fibroblasts, known as fibroblast progenitor cells^[^ ^15^ ^]^; *HAS1* ^+^ fibroblasts, exhibiting inflammation‐related features and elevated iCAFs scores; *LRRC15* ^+^ and *MME* ^+^ fibroblasts represented CAF subtypes, with *LRRC15* ^+^ CAFs characterized by high myCAF score and notably associated with poor prognosis in PDAC (Figures S3G–J, Supporting Information). We observed a positive correlation between the infiltration proportion of *PLXDC1* ^+^ TPSCs and *LRRC15* ^+^ myCAFs in 1169 PDAC samples (Figure 3I). Additionally, *LRRC15* ^+^ myCAFs exhibited a significantly high extent of PSC‐derived CAF characteristics and ECM features (Figure S3K, Supporting Information), consistent with *PLXDC1* ^+^ TPSCs. These findings provide additional evidence supporting the potentially malignant role of *PLXDC1* ^+^ TPSCs in the transition into *LRRC15* ^+^ myCAFs in the PDAC TME.

### 2.4 Differential Spatial Distribution of PSC Subclusters Exhibits Heterogeneous Niches

**Figure 4:**
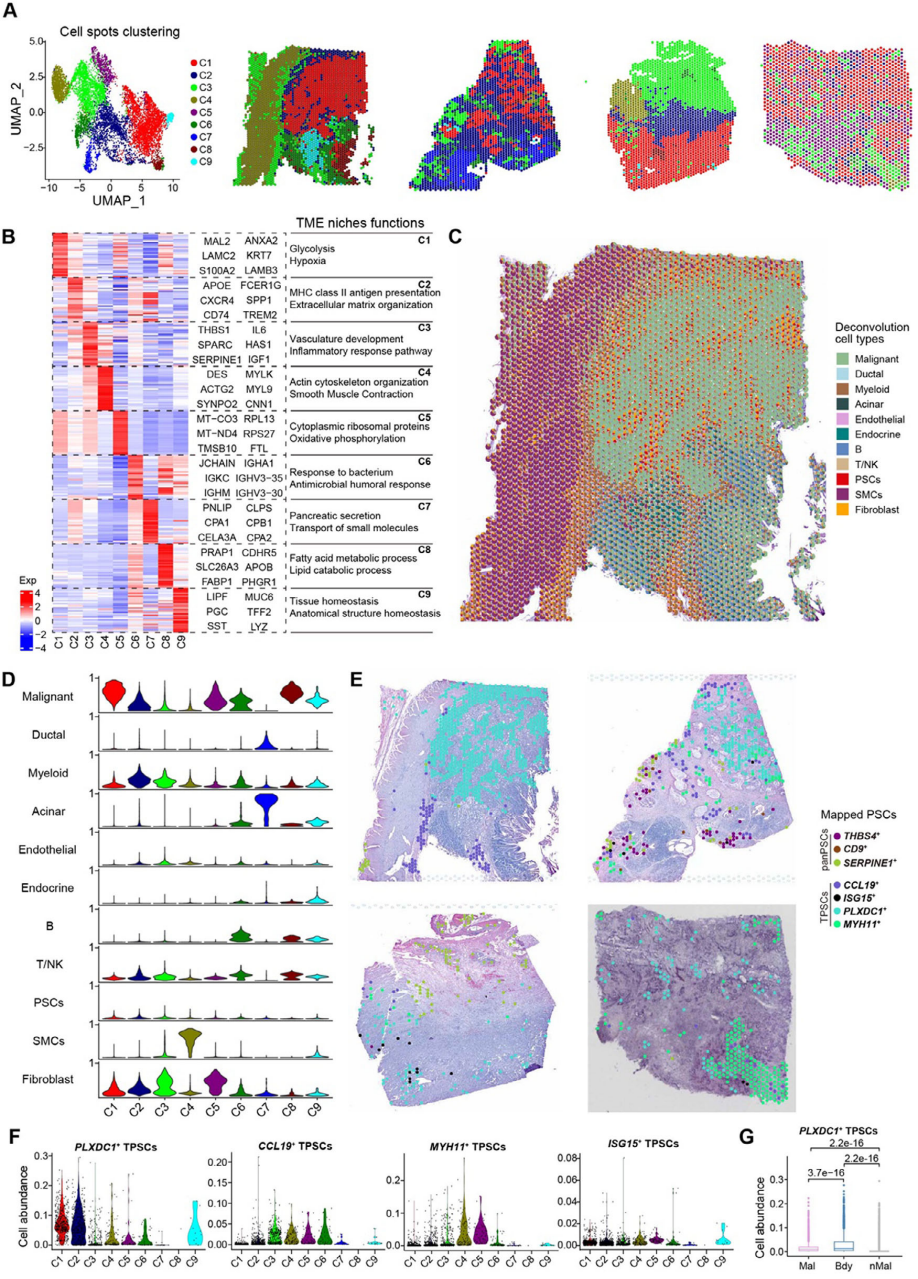
Differential spatial distribution of PSC subclusters exhibits functionally heterogeneous niches. A) UMAP plots of 9486 spots from four PDAC slides, showing nine major TME niches. The distribution of each TME niche on HE slides is shown. B) Heatmap showing the differentially expressed genes in each TME niche. Exp, z‐score normalized mean expression. Representative genes and Gene Ontology (GO) terms enriched in each TME niche are shown. Statistical analysis of enriched GO terms was performed using the hypergeometric test with adjusted Benjamini‐Hochberg *P*‐values. C) HE images with pie charts in each spot colored by annotation of major cell types showing the cellular composition in the PDAC TME. D) Violin plots showing the abundance of 11 major cell subtypes in each TME niche. E) Spatial mapping of PSC subclusters across tumor sections (*n* = 4) using Cell2location. Spots were filtered based on an estimated abundance of PSCs above 10%, and the specific subcluster of PSCs assigned was determined based on the highest proportion among the PSC subclusters. F) Violin plots showing the cell abundance of four TPSC subclusters in each TME niche. G) Box plot showing the abundance of *PLXDC1* ^+^ TPSC cell subcluster across different spatial regions including the malignant region (Mal), tumor boundary (Bdy), and nonmalignant region (nMal). The corresponding hematoxylin and eosin‐stained images were used as a background to provide a spatial location reference in C and E.

To characterize the spatial distribution of PSC subclusters with distinct transcriptional states and their interaction with the neighboring TME, we analyzed spatial transcriptomics (ST) of four PDAC tumor slides from previous studies.^[^ ^24^, ^25^ ^]^ We identified nine clusters shared across these slides, each occupying a spatial niche associated with specific functional pathways (**Figure** **4**A,B; Figure S4A, Supporting Information). For example, cluster 1 located in the tumor core was related to glycolysis and hypoxia, whereas cluster 7 in the normal acinar and ductal tissues was related to pancreatic secretion and small molecule transport. Cluster 2 in the interface region between the tumor and adjacent stromal tissue was related to antigen presentation and ECM organization, whereas clusters 3 and 4 in the stromal region were associated with vasculature development and smooth muscle contraction, respectively. Then, cellular composition within each spot (Figure 4C; Figure S4B, Supporting Information) was evaluated using Cell2location^[^ ^26^ ^]^ based on pre‐annotated scRNA‐seq reference (Figure 1B), consistent with TME niches functions and histopathological images from hematoxylin and eosin (HE) staining. Mapping cell abundance of different PSC subclusters revealed *PLXDC1* ^+^ TPSCs predominantly located in neighboring clusters 1 and 2, enriched at the tumor edge (Figure 4D–F). Additionally, by dividing ST slides into malignant region, tumor boundary, and nonmalignant region^[^ ^27^ ^]^ (Figure S4C, Supporting Information), *PLXDC1* ^+^ TPSCs were significantly enriched at the tumor boundary (Figure 4G). Other PSC clusters display distinct distributions: *CC19* ^+^ TPSCs tended to localize around the stromal or immune‐enriched region; *MYH11* ^+^ TPSCs in the stromal wall characteristic of SMCs; *SERPINE1* ^+^ CPSCs enriched in stromal region with a normal vascular signature; and the remaining CPSC subclusters showing low abundance, mainly in normal acinar and ductal region in PDAC ST slides (Figure 4B,F; Figure S4D, Supporting Information). Collectively, our results suggest the differential spatial distribution of PSC subclusters associated with different functions and cellular composition of spatial niches.

### 2.5 Co‐Localization of *PLXDC1* ^+^ TPSCs with *LRRC15* ^+^ myCAFs and *SPP1* ^+^ Macrophages Around Basal‐Like Tumors

**Figure 5:**
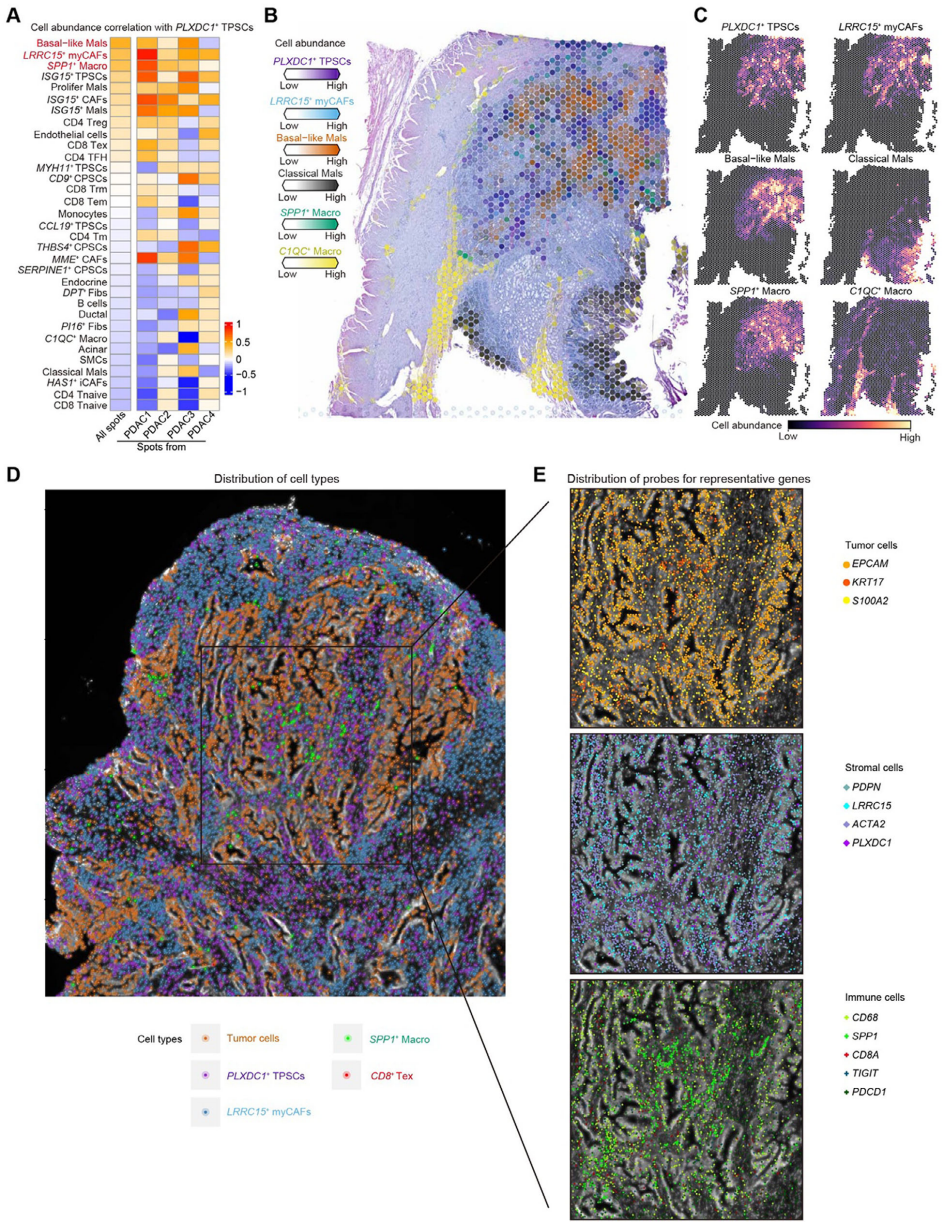
Co‐localization of PLXDC1+ TPSCs with LRRC15+ myCAFs and SPP1+ macrophages around basal‐like tumor. A) Heatmap showing the pairwise Pearson correlation between the proportions of infiltrating *PLXDC1* ^+^ TPSCs and the proportions of other cell clusters present within each spatial spot. The cellular composition within each spatial spot was assessed using Cell2location.^[^ ^26^ ^]^ B) Estimated cell abundances (color intensity) of different cell types (color) across each spatial spot. C) Estimated cell abundance (color intensity) of six representative cell clusters shown separately. D, E) Spatial distribution of five cell types (colors) (D) and detected probes for representative genes (colors) (E) on PDAC slices, information from ISS data. The corresponding cell segment image was used as a background to provide a spatial cell location reference. Corresponding hematoxylin and eosin‐stained images were used as a background to provide a spatial location reference in B, C, D, and E.

To gain insights into the interactions between distinct PSC subclusters and other cell types in the TME at the spatial level, we profiled 34 subpopulations of cell types in the TME (Figure S5A–G, Supporting Information). Next, using these more refined subgroups as references, we used Cell2location to assess the cellular composition within each spatial spot and calculated the pairwise Pearson correlations within the infiltrations of these cell clusters. The results revealed different PSC subclusters with corresponding co‐infiltrating TME cell types (Figure S5H, Supporting Information). For example, *THB4* ^+^ CPSCs and *CD9* ^+^ CPSCs tended to co‐infiltrate with acinar and ductal cells in nonmalignant regions (Figure 4B,C; Figure S4B–D and S5H, Supporting Information). The abundance of *SERPINE1* ^+^ vascular CPSCs positively correlated with *HAS1* ^+^ iCAFs and endothelial cells in regions associated with inflammation and vasculature (Figure 4B,C; Figure S4B,D and S5H, Supporting Information). Notably, *PLXDC1* ^+^ TPSCs localized closely with poor prognosis–related *LRRC15* ^+^ myCAFs,^[^ ^6^ ^]^ *SPP1* ^+^ macrophages,^[^ ^28^, ^29^ ^]^ and basal‐like malignant cells (Mals),^[^ ^30^, ^31^, ^32^ ^]^ forming a hostile niche around the tumor boundary (**Figure** **5**A–C; Figure S5H,I, Supporting Information). Furthermore, we observed that *PLXDC1* ^+^ TPSCs were negatively or nonsignificantly correlated with naïve cells, effector memory cells, and resident memory of *CD4* ^+^ and *CD8* ^+^ T cells, but were positively correlated with regulatory T (Treg) and exhausted T (Tex) cells (Figure 5A). PDAC is recognized as a cold tumor and is distinguished by the exclusion of T cells from the malignant tumor core, whereas Treg and Tex cells are predominantly enriched near the tumor boundary (Figures S5J,K, Supporting Information). Spatial plots further revealed that Tex cells were excluded from areas surrounding basal‐like Mals and co‐located with *PLXDC1* ^+^ TPSCs (Figure S5L,M, Supporting Information). To validate the *PLXDC1* ^+^ TPSC‐associated niche, we selected genes with specific expression profiles to represent their individual components. We specifically chose *EPCAM*, *KRT17*, and *S100A2* to represent basal‐like Mals^[^ ^32^ ^]^; *PDPN* and *LRRC15* for *LRRC15* ^+^ myCAFs^[^ ^6^ ^]^; *ACTA2* and *PLXDC1* for *PLXDC1* ^+^ TPSCs; *CD68* and *SPP1* for *SPP1* ^+^ macrophages^[^ ^28^ ^]^; and *CD8A*, *TIGIT*, and *PDCD1* for CD8 Tex cells^[^ ^33^ ^]^ (Figure S5N, Supporting Information). Subsequently, we conducted ISS using human PDAC tissue slides to confirm the hostile niche with *PLXDC1* ^+^ TPSCs, *LRRC15* ^+^ myCAFs, and *SPP1* ^+^ macrophages around basal‐like Mals to exclude T cells (Figure 5D,E; Figure S5O,P, Supporting Information). Our findings suggested that *PLXDC1* ^+^ TPSC–related niche around the tumor boundary may exclude T cell infiltration and promote the exhaustion of its neighboring T cells.

### 2.6 *PLXDC1^+^* TPSCs Promote a Desmoplastic and Immunosuppressive Niche Predicting Poor Immunotherapy Efficacy

**Figure 6:**
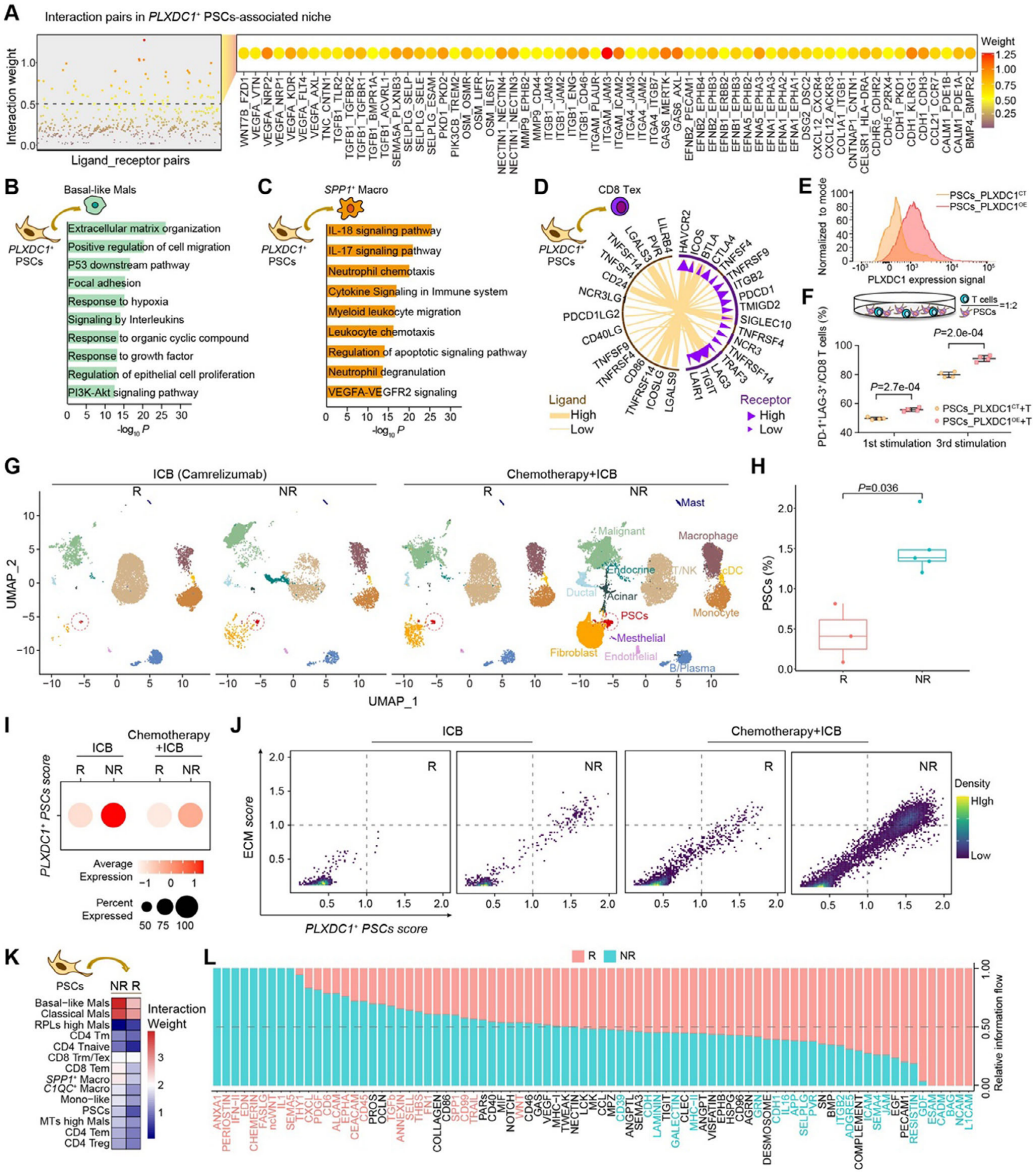
PLXDC1+ TPSCs promote a desmoplastic and immunosuppressive niche that predicts poor immunotherapy efficacy. A) Top‐ranking ligand‐receptor interactions in the relevant spatial niches of *PLXDC1* ^+^ TPSCs. The color intensity represents the strength of the interactions. B, C) Representative Gene Ontology (GO) enrichment of predicted target genes expressed in basal‐like malignant cells (B) and *SPP1* ^+^ macrophages, (C) resulting from interactions with *PLXDC1* ^+^ TPSCs. The hypergeometric test was performed using the Benjamini‐Hochberg procedure with adjusted *P*‐values. D) Interactions of immune checkpoint ligands and receptors between *PLXDC1* ^+^ TPSCs and exhausted CD8 T cells. The width of the lines and arrows indicates ligand and receptor expression, respectively. E) The PLXDC1 expression level in the culture of PLXDC1‐control (CT) PSCs or PLXDC1‐overexpressed (OE) PSCs. F) Dot plot with error bars showing the proportion of PD‐1^+^LAG3^+^CD8^+^ T cells within the total CD8^+^ T cells in co‐cultures of PLXDC1^CT^ PSCs or PLXDC1^OE^ PSCs with T cells. Data represent mean ± SD. Statistical difference was determined by a two‐tailed unpaired Student's t‐test. G) UMAP plots display the major cell types in PDAC patients before ICB or chemotherapy combined with ICB treatment. R, responders; NR, non‐responders. Each dot represents one cell. Cells are color‐coded by cell clusters accordingly. H) Box plots showing differential percentages of PSCs between responders (R) and nonresponders (NR). Box middle lines, median; box limits, upper and lower quartiles; box whiskers, 1.53 the interquartile range. Statistical differences were determined using the Wilcoxon rank‐sum test. I) Dot plots showing the average expression (dot color) and percentage of cells expressed (dot size) of the *PLXDC1* ^+^ PSCs signatures score for responders (R) and non‐responders (NR) with ICB or chemotherapy combined with ICB treatment. J) The expression diagram of *PLXDC1* ^+^ PSCs signatures score (x‐axis) and ECM signatures score (y‐axis) in PSCs from responders (R) and nonresponders (NR). K) The interaction weight between PSCs and other cell clusters in responders (R) and non‐responders (NR). L) Significantly enriched interaction pathways were observed separately in responders (R) and nonresponders (NR).

To elucidate cellular communication within the *PLXDC1* ^+^ TPSC‐associated niche, we analyzed cell‐cell interaction using NicheNet.^[^ ^34^ ^]^ Top‐ranking ligand‐receptor pairs were identified, covering ECM interactions (e.g., MMP9‐CD44, COL1A1‐ITGB1, and ITGB1/ITGA4‐JAMs), cytokine interactions (e.g., CXCL12‐CXCR4, CCL21‐CCR7, and VEGFA‐NRPs), and interactions known to facilitate tumor cell proliferation and migration (e.g., CDH1‐CDHRs, EFNA/Bs‐EPHA/Bs, and TGFB1‐TGFBRs) (**Figure** **6**A). Further, we studied the regulatory roles of *PLXDC1* ^+^ TPSCs on basal‐like Mals and *SPP1* ^+^ macrophages within the niche. *PLXDC1* ^+^ TPSCs interacted with basal‐like Mals through ligands (e.g., TGFB1, TGFB3), promoting biological processes such as ECM organization, hypoxia, and epithelial cell proliferation (Figure 6B; Figure S6A,B, Supporting Information). Similarly, *PLXDC1* ^+^ TPSCs interact with *SPP1* ^+^ macrophages via ligands such as CCL2 and ANXA1, fostering immunosuppressive functions including the IL‐18/IL‐17 and VEGFA‐VEGFR2 signaling pathways (Figure 6C; Figure S6C, Supporting Information). Consequently, these interactions collectively contributed to a desmoplastic and immunosuppressive niche.

To further investigate the relationship between *PLXDC1* ^+^ TPSC‐associated immunosuppressive niche and the exclusion of T cells around the tumor boundary, we analyzed interactions between *PLXDC1* ^+^ TPSCs and CD8 Tex cells. Multiple immune checkpoint ligand‐receptor interactions, including LGALS3‐LAG3, LGALS9‐HAVCR2, and CD24‐SIGLEC10 were identified (Figure 6D), suggesting a potential role of *PLXDC1* ^+^ TPSCs in regulating T‐cell exhaustion. Then we constructed PLXDC1‐overexpressed (OE) PSCs from CD36^+^ quiescent PSCs cell line and performed a co‐culture assay^[^ ^35^ ^]^ with T cells (Figure 6E). The results showed that co‐culturing PLXDC1‐OE PSCs with T cells significantly increased the percentage of PD‐1^+^LAG3^+^ exhausted CD8 T cells compared to the control group (Figure 6F; Figure S6D, Supporting Information), confirming that *PLXDC1* ^+^ TPSCs promote CD8 T cell exhaustion. Additionally, we conducted a PDAC cohort of 8 patients with immune‐checkpoint blockade (ICB) treatment (camrelizumab, a PD‐1 inhibitor) or combined treatment (abraxane plus gemcitabine chemotherapy along with ICB) and performed scRNA‐seq on the pre‐treatment tumor biopsies to explore the association between *PLXDC1* ^+^ TPSCs and ICB effectiveness. We first verified that *PLXDC1* ^+^ TPSCs signature scores were negatively associated with cytotoxic levels and positively associated with exhaustion levels in scRNA data from these eight patients (Figure S6E, Supporting Information). Then, patients were classified as responders (*n* = 3) or nonresponders (*n* = 5) according to the universal RECIST criteria.^[^ ^36^ ^]^ We profiled 14 cell types for ICB‐treated responders and non‐responders (Figure 6G; Figure S6F–I, Supporting Information). Accordingly, responders demonstrated higher cytotoxic levels while nonresponders exhibited elevated exhaustion levels in their T cells (Figure S6J, Supporting Information). Subsequently, we assessed the correlation between *PLXDC1* ^+^ TPSCs and the outcomes of ICB treatment. We found higher PSC infiltration in non‐responders versus responders (Figure 6H). Additionally, *PLXDC1* ^+^ TPSC signature scores positively correlated with ECM scores, particularly enriched in non‐responders (Figure 6I,J; Figure S6K, Supporting Information), suggesting *PLXDC1* ^+^ TPSCs may predict poor immunotherapy efficacy. Furthermore, cell‐cell interaction analysis revealed stronger TPSCs interactions with other cell clusters in non‐responders, including malignant tumor cells, *SPP1* ^+^ macrophages, and CD8 Tem/Trm/Tex cells (Figure 6K; Figure S6L, Supporting Information). Moreover, enriched interaction pathways like TGFB, ANXA1, and CXCL were predominantly observed in non‐responders, aligning with the interactions enriched in the *PLXDC1* ^+^ TPSC‐associated niche, thereby confirming the potential of *PLXDC1* ^+^ TPSC in regulating immune suppression (Figure 6L).

Our study provides a comprehensive characterization of PSCs, distinct from and complementary to *PDPN* ^+^ fibroblasts within the PDAC TME, unveiling their activation remodeling and their heterogeneity in molecular profiles, spatial distribution, and functional roles among different PSC subtypes. Collectively, these findings provide insights into potential future strategies to develop therapies that target PSCs.

## 3 Discussion

Previous studies on PSCs' activation and their roles in tumorigenesis and drug resistance mainly treated total PSCs as one type. Thus, this limited the study of PSCs' molecular diversity and regulatory mechanisms in the PDAC TME. Here, we investigated the complexity of PSCs in PDACs and provided single‐cell and spatial atlases to characterize their diversity and functions.

In a healthy pancreas, quiescent PSCs have vitamin A lipid droplets and are around acinar cells, small pancreatic ducts, pancreatic islets, and blood vessels to maintain tissue homeostasis and response to injury.^[^ ^4^, ^23^ ^]^ Activated PSCs have a myofibroblast‐like phenotype under inflammatory or pathological conditions.^[^ ^36^ ^]^ Additionally, growth factors, cytokines, and stressors are involved in PSC activation.^[^ ^10^ ^]^ However, the transcriptional and epigenetic regulation of PSCs in PDAC tumorigenesis is not fully understood. We isolated quiescent and activated PSCs from adjacent normal and PDAC tissues to profile their transcriptomics and epigenetic reprogramming. We found that stress‐ (e.g., *JUNB*, *ATF3*) and hypoxia‐related (e.g., *HIF1A*, *TWIST2*) TFs regulate the activation process. These findings imply that hypoxia‐related TFs in the TME could be the key regulator in PSCs activation. The mechanism might require further experimental validation.

PSCs exhibit remarkable plasticity in vitro, capable of differentiating into various cell types under specific conditions, including insulin‐secreting cells, adipocyte‐like cells, osteocyte‐like cells, and fibroblasts.^[^ ^37^ ^]^ Activated PSCs also present characteristics of other cell types,^[^ ^11^, ^38^ ^]^ like GFAP for glial cells, desmin for muscle cells, and vimentin for fibroblasts and endothelial cells. While in vitro studies have demonstrated that PSCs possess the capacity to differentiate into different CAFs phenotypes with local‐activation stimuli,^[^ ^39^ ^]^ in vivo experiments by Sherman et al. utilizing a Fabp4‐Cre tracing mouse model revealed that PSCs can only become a small subset of *PDPN* ^+^ CAFs.^[^ ^5^ ^]^ Our findings further corroborate the distinct molecular profiles of PSCs compared to *PDPN* ^+^ fibroblasts within PDAC TME, affirming their independent nature. Notably, our study delineates three distinct differentiation paths from CPSCs to TPSCs: *PLXDC1* ^+^, *MYH11* ^+^, and *CCL19* ^+^ TPSCs, which exhibited myofibroblast, SMC‐like, and inflammation‐related phenotypes, respectively. Although *PLXDC1* ^+^ TPSCs and *LRRC15* ^+^ myCAFs exhibit differential gene expression profiles, they co‐localize spatially, both displaying a myofibroblast phenotype and sharing functional similarities in predicting poor ICB therapy response. This observation also implies that *LRRC15* ^+^ myCAFs may have two distinct sources: one originating from *DPT* ^+^ fibro‐progenitor cells,^[^ ^15^, ^40^ ^]^ while the other could potentially be derived, to some extent, from *PLXDC1* ^+^ TPSCs, thus supplementing the *LRRC15* ^+^ myCAFs pool.

Activated PSCs are associated with immunosuppression and an increase in regulatory T cells, M2 macrophages, and myeloid‐derived suppressor cells when pancreatic cancer cells are co‐injected with PSCs in a mouse model.^[^ ^41^ ^]^ Activated PSCs also can reduce the infiltration of cytotoxic T cells into the PDAC TME, interfering with the efficacy of immunotherapy in patients with PDAC.^[^ ^42^ ^]^ However, the interactions between PSCs and specific types of immune cells in the TME as well as their regulatory mechanisms remain unclear. This study demonstrated that *PLXDC1* ^+^ TPSCs could localize closely with basal‐like Mals and *SPP1* ^+^ macrophages around the tumor boundary to generate a desmoplastic and immune‐suppressive niche. *PLXDC1* ^+^ TPSCs not only interacted with basal‐like Mals to promote a desmoplastic microenvironment but also expressed high levels of ligands such as CCL2, ANXA1, and IL34, interacted with *SPP1* ^+^ macrophages, and regulated their immunosuppressive functions. The *PLXDC1* ^+^ TPSC‐related spatial niche around the tumor boundary acted as a barrier to T cells, and *PLXDC1* ^+^ TPSCs interacted with T cells via immune checkpoint receptors, thereby promoting T cell exhaustion. Furthermore, we demonstrated that *PLXDC1* ^+^ TPSCs linked to immunotherapy resistance in our in‐house ICB treatment PDAC cohorts, suggesting that PDAC patients may benefit from *PLXDC1* ^+^ TPSC inhibitors combined with immunotherapy.

Despite the comprehensive multi‐omics approach and the novel insights into *PLXDC1* ^+^ PSCs in the tumor microenvironment, our study has several limitations. First, while we validated the immunosuppressive role of *PLXDC1* ^+^ PSCs through co‐culture assays, phenotypic confirmation at the protein level and functional validation through loss‐of‐function studies in animal models remain to be addressed. Second, the differentiation towards immunosuppressive pathways was inferred based on predictive models and co‐culture results, lacking direct in vivo evidence. Finally, the generalizability of our findings may be limited by the clinical sample size and the reliance on a single ICB‐treated PDAC cohort. Regarding the predictive liability of *PLXDC1* ^+^ TPSC for immunotherapy efficacy, further validation in larger patient cohorts is needed, and the underlying complex regulatory mechanisms remain to be explored. These limitations highlight avenues for future research, including mechanistic studies and validation in larger, independent cohorts.

In summary, we systematically characterized the activation remodeling, heterogeneity, spatial distribution, and functions of PSCs within the PDAC TME, providing PSC‐targeted therapies with immunotherapy in the future.

## 4 Experimental Section

### Human Specimens for Fluorescence‐Activated Cell Sorting (FACS) and ISS

Pancreatic cancer tissue samples were acquired from patients undergoing pancreatic resection without prior chemotherapy or targeted systemic therapy at the Department of Pancreatic‐Biliary Surgery, Changzheng Hospital, Naval Medical University. Subsequently, the PDAC tissue samples were processed for downstream FACS experiments or freshly frozen and embedded in an optimal cutting temperature (OCT) compound. The OCT‐embedded tissue was then sectioned into 10‐µm‐thick slices for ISS experiments.

Before FACS experiments, the collected surgical tissues were processed as follows. The tumor tissues were preserved in complete RPMI 1640 medium containing 1x protease inhibitor (Solarbio, P6730), which included 10% FBS, 100  U mL^−1^ penicillin, and 100 mg mL^−1^ streptomycin, while adjacent normal tissues were stored in complete RPMI 1640 medium with 2x protease inhibitor (Solarbio, P6730). Both types of tissues were maintained on ice before subsequent processing. The samples were rinsed with ice‐cold PBS and then cut into approximately 2 mm‐sized pieces. Each sample was enzymatically digested using a mixture consisting of 1 mg mL^−1^ Trypsin inhibitor (Sigma, T6522), 0.82 mg mL^−1^ Dispase (Sigma, D4693), collagenase VIII at 1 mg mL^−1^, and DNase I at 0.15  mg mL^−1^ in 10 mL complete RPMI 1640 medium. Tumor tissues were dissociated at 37  °C with a shaking speed of 200 r.p.m for approximately 50 min. The dissociated cells were collected at intervals of 25 min to enhance cell yield and viability. For adjacent normal tissues, minced tissues were incubated with the same digestion buffer at 37 °C with a shaking speed of 200 r.p.m for approximately 30 min. The dissociated cells were collected at intervals of 15 min. Following digestion, vigorous shaking was performed to mechanically separate the cells, and then 10 mL of complete RPMI 1640 medium was added to terminate the digestion process. Red blood cells were removed by incubating with 2 mL of ACK lysing buffer (Thermofisher, A1049201) for 5 min on ice. Then, the dissociated cells were used for FACS experiments.

### Human Specimens in ICB Treatment Cohort

In this study, eligible PDAC patients were recruited at Ruijin Hospital, in Shanghai, China. Patients enrolled in this study were those who had histologically confirmed advanced PDAC and received ICB therapy (Camrelizumab, a PD‐1 inhibitor) or combined treatment therapy (abraxane plus gemcitabine (AG) along with ICB). The tumor biopsy tissues were collected from PDAC patients before treatment with informed written consent. Each tumor was punctured by two or three needles.

The collected tumor tissues were stored with 1x protease inhibitor (Solarbio, P6730) in complete RPMI 1640 medium (containing 10% FBS, 100 U mL^−1^ penicillin, and 100 mg mL^−1^ streptomycin) and were kept on ice before subsequent processing. Samples were flushed with ice‐cold PBS and cut into pieces of approximately 2 mm in length. Each sample was then enzymatically digested with 1% Trypsin inhibitor (Sigma, T6522), 0.05% Dispase (Sigma, D4693), collagenase VIII at 1 mg mL^−1,^ and DNase I at 0.15 mg mL^−1^ in 3 mL complete RPMI 1640 medium for 15 min at 37 °C. After digestion, 21‐gauge syringes were used to dissociate cells mechanically and then 4 mL of complete RPMI 1640 medium was added for termination of digestion. Red blood cells were removed through 2ml ACK lysing buffer (Thermofisher, A1049201) for 5 min on ice. After washing twice with 1x PBS (Corning), the cell pellets were re‐suspended in the single‐cell buffer. Subsequently, the cell suspension was filtered through 70 µm cell strainer (BD) and then 1% protease inhibitor was added. Freshly prepared cell suspensions were ready for constructing single‐cell RNA‐seq libraries by using the Chromium Next GEM Single Cell 5′ Kit v2 from 10x Genomics, following the manufacturer's instructions. Then, the single‐cell RNA libraries were sequenced by an Illumina HiSeq X‐Ten sequencer with 150 bp paired‐end reads.

### Single‐Cell RNA Sequencing (scRNA‐seq) Data Analysis

In this study, the scRNA‐seq data was obtained for 24 primary PDAC tumors and 11 normal pancreas samples from non‐pancreatic tumor or nonmalignant pancreatic tumor patients, originally published by Peng et al.^[^ ^43^ ^]^ Additionally, scRNA‐seq data from 8 PDAC tumors within an in‐house ICB cohort were also utilized. Subsequently, a standardized analysis of scRNA‐seq data was conducted using the R package Seurat v5.0.0.^[^ ^44^ ^]^ Cells were filtered out with fewer than 300 or more than 6000 detected genes, as well as those with fewer than 500 or more than 50000 UMI counts, and cells containing over 15% mitochondrial gene counts. Additionally, the Python package Scrublet v0.2.3 was utilized^[^ ^45^ ^]^ with default parameters to eliminate predicted doublets. After quality control, a total of 139463 cells remained available for downstream analysis.

The standard Seurat has followed workflow for dimension reduction and unsupervised clustering. In brief, the UMI was log‐normalized counts representing gene expression and identified the top 2000 highly variable genes (HVGs) using the FindVariableFeatures function. Subsequently, the data and performed principal component analysis (PCA) were scaled based on the expression of HVGs. Utilizing the top 20 principal components (PCs), clusters were identified with the “FindNeighbors” and “FindCluster” functions in Seurat and visualized using the uniform manifold approximation and projection (UMAP) algorithm. After the first‐round of unsupervised clustering, major cell types were annotated, including epithelial cells (malignant cells, ductal cells, endocrine cells, and acinar cells), immune cells (T cells/natural killer cells, B cells/plasma cells, and myeloid cells), and stromal cells (fibroblasts, pancreatic stellate cells (PSCs), smooth muscle cells (SMCs), and endothelial cells), based on known gene signatures (Table S1, Supporting Information). To identify subclusters within the PSCs cell type, a second round of unsupervised clustering for PSCs was conducted, following the same procedure as described above. Additionally, the R package Harmony v0.1.1^[^ ^19^ ^]^ or reciprocal PCA (RPCA)‐based integration^[^ ^20^ ^]^ to eliminate batch effects across different samples was employed.

### Marker Gene Identification for Individual Cell Clusters

Cell cluster‐specific marker genes were identified using the “FindAllMarkers” function within the Seurat package. In summary, each cell cluster was compared against all other clusters with the following parameters: min.pct = 0.2, logfc.threshold = 0.25, only.pos = T. *P*‐values were obtained through the Wilcoxon rank‐sum test and subsequently adjusted using Bonferroni correction.

### Tissue Distribution of PSCs Subclusters

To evaluate the enrichment preferences of individual PSCs subclusters in either control pancreatic tissue or PDAC tumors, the observed‐to‐expected cell number ratio (R_O/E_) was calculated. The expected cell numbers for each combination of cell clusters and tissues were determined using the chi‐square test. A PSCs subcluster was considered enriched in a specific tissue if the R_O/E_ ratio exceeded 1. This analysis allowed them to quantify the degree of tissue specificity for each PSCs subcluster.

### Estimation of Cell Clusters Fractions in Bulk Tissues

To characterize the infiltration of cell clusters within PDAC tissues from bulk RNA‐seq and microarray data, the CIBERSORTx^[^ ^21^ ^]^ tool was employed. This involved the generation of a reference feature matrix from the scRNA data, which was subsequently employed to estimate the proportions of cell clusters in the bulk transcriptome data, based on the constructed cell cluster reference. During the creation of the feature matrix, CIBERSORTx was executed with quartile normalization disabled for bulk transcriptome datasets, utilizing 500 permutations for the permutation parameter, while all other parameters were maintained at their default settings.

### Calculation of Gene Signature Enrichment Scores

The gene sets used to calculate functional scores across various clusters, such as PSCs clusters (HIF1A targets score, hypoxia score, PSCs‐derived CAFs score, and PSCs‐derived CAFs‐associated ECM score), fibroblasts clusters (iCAFs score and myCAFs score), and malignant cells clusters (basal‐like score, classical score, proliferating score, interferon score, cancer_ECM score, and hypoxia score), were obtained from previously published papers^[^ ^2^, ^5^, ^6^, ^32^, ^46^ ^]^ and are summarized in Table S6 (Supporting Information). To calculate the enrichment scores for these gene sets, a methodology as previously described was followed (https://www.github.com/cssmillie/ulcerative_colitis). In brief, the gene signature score for each individual cell was determined by computing the mean scaled expression across all genes within a particular gene set.

### Single Cell Trajectory Analysis

RNA velocity estimates gene splicing and degradation rates and infers dynamic cell trajectories by calculating the abundance of nascent pre‐mRNA (unspliced) and mature mRNA (spliced). To elucidate the differentiation trajectory of PSCs, the velocyto.py software package was utilized (https://velocyto.org/velocyto.py/, v0.17.16) to compute read counts for RNA splicing and unspliced transcripts from the scRNA‐seq data. Subsequently, these data were input into the scVelo software package (v0.2.4) to calculate RNA velocity values for each gene in each cell. The resulting RNA velocity vectors were embedded in a UMAP plot for visualization. A diffusion map algorithm was also applied^[^ ^47^ ^]^ to confirm the trajectory result from the scVelo. In brief, the PSCs subclusters expression matrix and the previously calculated PCs matrix were fed into the scanpy pipeline (v 1.8.1). A neighborhood graph based on the top 20 PCs was constructed using the “scanpy.pp.neighbors” function. Given a neighborhood map, a diffusion map was constructed using the “scanpy.tl.diffmap” function. The first two diffuse components (DCs) were used for visualization. Diffusion pseudotime was calculated using “scanpy.tl.dpt” function depending on the positions of less differentiated cells (*THBS4* ^+^ PSCs) on the diffusion map. PHATE was also performed,^[^ ^48^ ^]^ a tool for visualizing high dimensional data based on a novel conceptual framework for learning and visualizing the manifold to preserve both local and global distances, to infer the differentiation states of PSCs subsets.

### Flow Cytometry

Dead cells were labeled with fixable viability stain 620 (BD Biosciences) and subsequently eliminated from the analysis. To prevent nonspecific binding to Fc receptors, CD16/32 antibody (Biolegend) was utilized as a blocking agent prior to surface staining. For surface staining, cells were suspended in 100 µL of FACS buffer (BD Biosciences) containing antibody cocktails and stained at 4 °C in the dark for 30 min. After staining, cells were washed before being analyzed using a FACSymphony (BD Bioscience) flow cytometer. The antibodies used for flow cytometry can be found in Table S2 (Supporting Information).

For cell sorting, PSCs isolated from tumor tissues and adjacent normal tissues of PDAC patients were sorted based on their characteristics as PDPN^−^lin^−^ (CD45^−^CD31^−^CD326^−^) CD140b^+^CD36^+^ live cells or PDPN^−^lin^−^ (CD45^−^CD31^−^CD326^−^) CD140b^+^CD36^−^CD90^+^ live cells. Data analysis was performed using FlowJo 10.81 software.

### Bulk mRNA‐seq and Data Analysis

For bulk mRNA‐seq, total RNA was isolated from FACS‐sorted cells using the Trizol reagent (Invitrogen). RNA quality was assessed using the RNA Nano 6000 Assay Kit on the Bioanalyzer 2100 system (Agilent Technologies, CA, USA). Subsequently, library construction commenced with mRNA purification from the total RNA through poly‐T oligo‐attached magnetic beads. This was followed by fragmentation and reverse transcription into cDNA. After cDNA blunt‐ending and 3′ end adenylation, adaptors with hairpin loop structures were ligated to facilitate hybridization. Amplification of cDNA fragments selectively targeted those within the 370–420 bp range using the AMPure XP system (Beckman Coulter, Beverly, USA) to generate the final library. The library was then sequenced on the Illumina NovaSeq 6000 platform.

For data analysis, sequencing reads were aligned to the human reference genome (GRCh38 version) using HISAT2 v2.1.0 (https://github.com/DaehwanKimLab/hisat2). Subsequently, StringTie v2.1.7 software was utilized (https://ccb.jhu.edu/software/stringtie/) to generate a gene counts matrix and quantify gene expression in transcripts per million (TPM). Differential expression analysis between two conditions was conducted following the standard DEseq2 v1.32.0 workflow. *P*‐values were computed using the Wald test and adjusted (padj) for multiple testing using the Benjamini and Hochberg methods. Differentially expressed genes (DEGs) meeting the criteria of |log2‐fold change| > 1 (equivalent to a 2‐fold change) and *P*‐value < 0.05 were visualized in a heatmap based on log‐transformed and scaled gene expression. PCA was performed on multiple datasets from different samples using the built‐in R function “prcomp”, and the first two PCs were presented in a scatter plot. The gene expression matrix is provided in Table S3 (Supporting Information).

### Bulk Assay for ATAC‐seq and Data Analysis

Bulk ATAC‐seq was performed as previously described.^[^ ^49^ ^]^ Briefly, a total of ∼1×10^5^ FACS‐sorted cells were prepared and washed with PBS, and subsequently resuspended in 100 µL of cold ATAC‐seq resuspension buffer (RSB; 10 mM Tris‐HCl pH 7.4, 10 mM NaCl, and 3 mM MgCl2 in water), supplemented with 0.1% NP40, 0.1% Tween‐20, and 0.01% digitonin. After gentle mixing and a 3‐minute incubation on ice, the cells were lysed. Next, 1 mL of ATAC‐seq RSB containing 0.1% Tween‐20 (without NP40 or digitonin) was added, and the mixture was inverted to ensure thorough mixing. The nuclei were pelleted by centrifugation at 500 RCF for 10 min in a pre‐chilled (4 °C) fixed‐angle centrifuge. The supernatant was carefully removed, and the nuclei were resuspended in 50 µL of a transposition mix (comprising 10 µL of 5× TTBL buffer, 5 µL of TTE Mix V50, 16.5 µL of PBS, 0.5 µL of 1% digitonin, 0.5 µL of 10% Tween‐20, and 17.5 µL of water). The transposition reactions were incubated at 37 °C for 30 min with shaking at 1000 RPM in a thermomixer. Subsequently, DNA was purified using a DNA Clean‐Up kit (Qiagen cat# 28306). DNA fragments underwent a 12‐cycle PCR to incorporate unique indices for sequencing and were purified using a PCR Purification Kit (Qiagen cat# 28106). Finally, the samples were subjected to paired‐end sequencing on a NovaSeq 6000 System (Illumina).

For data analysis, raw sequences were processed using fastqc v0.11.9 and cutadapt v3.4 to eliminate adapter sequences and low‐quality sequences. Subsequently, the clean reads were aligned to the human reference genome (GRCh38 version) using bowtie2 v2.2.5 with default parameters, and only uniquely and properly paired mapping reads were retained. After alignment, sequences mapping to mitochondrial DNA and duplicate sequences were filtered out, resulting in the final BAM files. These BAM files were then transformed into bigWig files using the bamCoverage tool from deepTools 3.5.4 (default settings), facilitating visualization with the Integrative Genomics Viewer (IGV) tool. Peak calling was performed using macs2 v2.2.7, applying a significance threshold of *p*‐value ≤ 0.05 and retaining peaks with a fold change ≥ 3. Identification of overlapping or differentially enriched peaks between two samples was accomplished using the bedtools intersect command within bedtools v2.25.0. In all, 12211 upregulated and 3895 downregulated peaks (open chromatin regions; OCRs) were found in activated PSCs. Differential peaks were further subjected to clustering and visualization using the “computeMatrix” and “plotHeatmap” programs in deepTools v3.5.1. Peak annotation was conducted using the “annotatePeaks.pl” program in homer v4.11.1 with default parameters. Additionally, enriched motifs within the peaks were identified using the “findMotifsGenome” program in homer v4.11.1, utilizing default parameters and testing for enrichment against a set of 413 known motifs available in the homer motif database. The peaks profile is provided in Table S4 (Supporting Information).

### Definition of Co‐Regulated Peaks

During PSC activation, changes occur at both the gene expression and chromatin accessibility levels, with peaks exhibiting concurrent alterations being of regulatory significance. To identify these regulatory peaks, the differentially enriched peaks defined during PSC activation using the “annotatePeaks.pl” tool in HOMER v4.11.1 were annotated. Peaks were defined as co‐regulated if they were annotated to differentially expressed genes and exhibited consistent directional changes. A total of 996 upregulated peaks in activated PSCs, associated with co‐upregulated gene expression, and 835 downregulated peaks in activated PSCs, associated with co‐downregulated gene expression, were identified.

### Spatial Transcriptomics (ST) Data Analysis

ST data for four slides of PDAC patients from a published paper was retrieved^[^ ^24^, ^25^ ^]^ and then followed the standard Seurat workflow for dimension reduction and unsupervised clustering of cell spots. This clustering was carried out with a resolution of 0.3, utilizing the first 25 PCs. To eliminate batch effects arising from different samples, the integrated Canonical Correlation Analysis (CCA) method within Seurat was applied. Subsequently, nine distinct clusters based on their marker genes, defined using the “FindAllMarkers” function in Seurat were identified and annotated. Spatial feature expression plots were generated using the “SpatialFeaturePlot” function in Seurat. The scores for signature modules were calculated as the mean expression levels of signature genes within each module.

### Definition of Distinct Regions in ST Slides

The in‐house developed method incorporated within the Cottrazm was used,^[^ ^27^ ^]^ a tool packaged within the R environment, to define the malignant (Mal) region, tumor boundary (Bdy), and nonmalignant (nMal) regions of the ST slides.

### Deconvolution of ST Spots

For cell type deconvolution, the cell2location v0.1.3 package was utilized^[^ ^26^ ^]^ along with a reference single‐cell dataset consisting of 7 subclusters of PSCs, 4 subclusters of malignant cells, 3 subclusters of macrophages, 8 subclusters of T cells, 6 subclusters of fibroblast cells, ductal cells, endocrine cells, acinar cells, B/Plasma cells, SMCs, and endothelial cells, totaling 34 cell types within PDAC tumors. The regression model was trained using default parameters to ensure optimal reconstruction accuracy. Ultimately, the cell2location model was established with the following parameters: N_cells_per_location = 20, detection_alpha = 20, max_epochs = 30 000. For nonnegative matrix factorization (NMF), we followed the default settings and selected a factor of six to strike a balance between interaction specificity and cell density. The abundances of cell types were calculated and exported back to Seurat. To visualize PSC‐enriched spots, spots based on the cell abundance above 10% were filtered. Subsequently, specific PSC subclusters based on the highest proportion among the PSC subclusters were assigned. Cell abundance in spatial coordinates was visualized using the “plot_spatial functions” of cell2location. This visualization considered only values from the posterior distribution that were greater than the 5th percentile, representing cell abundance values that the model had high confidence in.

### Functional Annotation of Genes

The Metascape tool (https://metascape.org/gp/index.html) was utilized for functional annotation of the identified marker genes or DEGs. In this analysis, the *P*‐value was defined as the probability of obtaining “*n*” or more pathway members, resulting in a cumulative hypergeometric distribution.

### Survival Analysis

Survival analysis was conducted using the R package survival v3.3.1. Overall survival (OS) data were obtained from 10 public PDAC datasets or the ICB‐treatment cohort (IMvigor210), as listed in the Data availability section. Hazard ratios (HR) were calculated using the Cox proportional hazards model, and 95% confidence intervals (CI) were reported. Kaplan–Meier survival curves were generated using the “survfit” function. To perform dichotomy of cell population infiltration or gene set enrichment scores, the “maxstat.test” function from the R package maxstat was employed. This function repeatedly tested all potential cutting points to find the maximum rank statistic, which was used to divide patients into two groups based on the selected maximum logarithm statistics. Kaplan–Meier survival curves were plotted using the “ggsurvplot” function and compared using a two‐sided log‐rank test. For gene set enrichment score calculation, the R package GSVA v1.42.0 from Bioconductor was utilized. The gene sets for analysis included the top 20 marker genes for each PSCs subcluster, the top 20 ligand‐receptor pairs for *PLXDC1* ^+^ PSCs‐associated ECM abundance, and the immune escape niche. Additionally, the correlation between the infiltration ratio of *LRRC15* ^+^ PSCs was validated, predicted by CIBERSORTx, and poor survival in the TCGA cohort.

### Cell‐Cell Interaction Analysis

The R package NicheNet v2.0.0^[^ ^34^ ^]^ was employed to identify the top ligand‐receptor interaction pairs in the context of *PLXDC1* ^+^ PSCs‐associated niche. To achieve this, spatial transcriptomic data was utilized from cell spots within the *PLXDC1* ^+^ PSCs‐associated niche as both the sender and receiver, while using other cell spots within the non‐*PLXDC1* ^+^ PSCs‐associated niche as reference. To account for ligand‐receptor interactions, only cell spots with gene expression levels above 10% were included, and ligand‐receptor interactions weighted above 0.5 were considered. Next, to further investigate the regulatory mechanisms underlying the interactions between *PLXDC1* ^+^ PSCs and basal‐like malignant cells, *SPP1* ^+^ macrophages, scRNA‐seq data was employed from relevant cell clusters and identified the top 50 ligands and the top 200 targets based on differentially expressed genes in both “sender cells” and “affected cells” for subsequent analysis of ligand‐target activity. To assess the regulatory potential of *PLXDC1* ^+^ PSCs (as sender cells) on basal‐like malignant cells (as affected cells), and *SPP1* ^+^ macrophages (as affected cells), classical malignant cells were used, *C1QC* ^+^ macrophages as reference cells. The ligand_activity_target_heatmap in Nichenet_output was used to visualize the regulatory activity of ligands, with activity scores ranging from 0 to 1.

To analyze the regulatory role of *PLXDC1* ^+^ PSCs on CD8 Tex, R toolkit iTALK was employed and assessed the interaction and expression of immune checkpoints between *PLXDC1* ^+^ PSCs and CD8 Tex based on the gene list provided within iTALK's checkpoint module.

To compare cell‐cell interactions of PSCs with other cell types between responders and non‐responders, R package Cellchat v1.5.0 was applied to evaluate the interaction weights of clusters, implemented with default parameters. Then, the compareInteractions() function was utilized to delineate differential cell‐cell interaction receptor‐ligand pairs between responders and non‐responders. Subsequently, rankNet() with the mode set to “comparison” was employed to identify significantly distinct interaction signaling pathways.

### ISS Assay Procedure

ISS was achieved using an improved ISS (IISS) technique, which incorporates an innovative probing and barcoding approach described by Tang et al. 2023^[^ ^50^ ^]^ to enable highly multiplexed in situ gene expression profiling. The labeling sequencing interrogation probe was conjugated with fluorophores, including Cy3, Texas Red, Cy5, or Alexa Fluor 750 (Life Technologies, Thermo Fisher Scientific, Shanghai, China).

In brief, 10‐µm‐thick fresh frozen tissue sections embedded in optimal cutting temperature (OCT) were firstly fixed using 4% (w/v) paraformaldehyde (Sigma, Shanghai, China) for 5 min. Subsequently, they were rinsed twice with phosphate‐buffered saline (PBS) treated with diethylpyrocarbonate (DEPC), and subjected to dehydration through an ethanol series. Following three washes with a washing buffer (SEERNA Bioscience, Xiamen, China), the sections underwent permeabilization in 0.1 m HCl at 37 °C for 5 min. Next, the sections were sequentially incubated with the following solutions: the hybridization mix, which included target probes labeled with unique barcodes for individual RNA molecules, at 37 °C for 4 h; the ligation mix at 37 °C for 30 min; the circularization mix of splint primers at 37 °C for 30 min and the amplification mix for rolling circle amplification (RCA) at 30 °C overnight. After each incubation step, sections were washed with Wash Buffer (SEERNA Bioscience, Xiamen, China) three times at room temperature. For sequencing, the sections were incubated in the ligation buffer containing anchor primers, label sequencing interrogation probes, and ligase at 30 °C for 45 min. Subsequently, they were mounted with SlowFade Gold Antifade Mountant (Thermo Fisher Scientific, Shanghai, China) containing 0.5 µg mL^−1^ DAPI (Sigma, Shanghai, China). Images were captured using the Leica DM6B microscope equipped with a DFC9000GT camera and a 20x objective lens. Following imaging, the sequencing probes were removed using a Stripping Buffer (SEERNA Bioscience, Xiamen, China). The sections then underwent three 5‐minute washes with Wash Buffer (SEERNA Bioscience, Xiamen, China) to prepare the sections for the next sequencing cycle. This same process was repeated for each cycle until the desired number of cycles were completed (up to four cycles), involving a sequence of ligation, imaging, and probe stripping. The TissUUmaps tool was utilized to visualize the expression of each gene on the section.

### The Culture of PSCs

The human PSCs cell line was purchased from ANWEISCI (Shanghai, China) and maintained in DMEM supplemented with 10% FBS and antibiotics (Invitrogen, Shanghai, China). These cells exhibited gene expression of CD36. To investigate whether hypoxic conditions could promote the activation of quiescent PSCs (*CD36* ^+^ PSCs), *CD36* ^+^ PSCs sorted from PDAC patients or the aforementioned *CD36* ^+^ PSCs cell line were initially cultured in a normoxic incubator (21% O2, 5% CO2) for 24 h, before being transferred to a hypoxic incubator (1% O2, 5% CO2). After 48 to 72 h, RNA was extracted from the cells for qPCR analysis.

### The Co‐Culture of PSCs with T Cells

The vector control and PLXDC1 expression plasmids were produced by Genomeditech (Shanghai, China). The human PSCs cell line was transfected with either the vector control or PLXDC1 expression plasmid using GMTrans Liposomal Transfection Reagent (Genomeditech, Shanghai, China), following the manufacturer's instructions. Stable clones overexpressing PLXDC1 or vector control were selected with 4 µg mL^−1^ puromycin (Beyotime, Shanghai, China) for 2 weeks. To obtain pan‐T cells, PBMCs from healthy individuals were cultured and induced with a human CD3/CD28/CD2 T cell activator (10970, STEMCELL) and 10 ng mL^−1^ IL‐2 for 2–3 days. Then cells were maintained in a resting state with only IL‐2 for another 3–4 days. PSCs with PLXDC1 overexpression or vector control were then inoculated into 6‐well plates at 70% confluence, 1 day prior. T cells were added when the PSCs reached 90% confluence (T cells: PSCs = 1:2) and co‐cultured. Cells were stimulated with a human CD3/CD28/CD2 T cell activator following the manufacturer's recommendations. Every 48 h, T cells were washed and restimulated with a fresh batch of CD3/CD28/CD2 T cell activator and also PSCs. After one or three stimulations, the expression of PD‐1 and LAG‐3 on CD8+ T cells was detected by flow cytometry.

### Statistical Analysis

The results were expressed as mean ± SD. Statistical analyses were performed using Prism 9.0 (GraphPad). The T‐test or Wilcoxon rank‐sum test (two‐tailed) was used to detect the statistical significance of the differences between the two groups. Any *P*‐values greater than 0.05 were considered statistically insignificant and were labeled accordingly in the corresponding Figures.

### Ethics Approval and Consent to Participate

All procedures performed with the use of samples obtained from PDAC patients without prior chemotherapy or targeted systemic therapy were approved by the Medical Ethics Committee of Naval Medical University (2018SL004). All procedures performed with the use of tumor biopsy tissues obtained from PDAC patients received ICB therapy approved by Ruijin Hospital Affiliated with Shanghai Jiao Tong University (RuijinH2020386). All the patients signed informed consent to publish. The research conformed to the principles of the Helsinki Declaration.

## Conflict of Interest

The authors declare no conflict of interest.

## Author Contributions

Y.D., Y.Z., and J.L. contributed equally to this work. Y.Y., D.Z., C.S., and S.C. conceived and supervised the project. Y.D., L.Z., and Y.Y. conducted analyses. Y.Z., J.L., Y.Z., C.S., D.Z., and J.Y. conducted the clinical cohort and collected samples. Y.Z., J.L., and J.W. performed the experiment. Y.D., H.A.R., J.L., C.S., D.Z., and Y.Y. were involved in the interpretation, and Y.D., Y.Z., and Y.Y. wrote the manuscript.

## Supporting information



Supporting Information

Supplementary TableS1

Supplementary TableS2

Supplementary TableS3

Supplementary TableS4

Supplementary TableS5

Supplementary TableS6

## Data Availability

This study incorporated publicly available single‐cell transcriptomics data obtained from the Genome Sequence Archive (https://ngdc.cncb.ac.cn/gsa‐human/) for PDAC under project code PRJCA001063.^[^ ^43^ ^]^ Spatial transcriptomics data for PDAC were acquired from the Gene Expression Omnibus (GEO) database with GSE211895^[^ ^24^ ^]^ and GSE203612.^[^ ^25^ ^]^ The survival analysis in this study encompassed 10 public PDAC datasets. Among these, there were three RNA‐seq datasets: TCGA‐PAAD (*n* = 185) from The Cancer Genome Atlas (TCGA) database, ICGC‐PACA‐CA (*n* = 234), and ICGC‐PACA‐UA (*n* = 91) from the International Cancer Genome Consortium (ICGC) database. Additionally, there were seven Affymetrix microarray datasets: GSE21501 (*n *= 132), GSE28735 (*n* = 45), GSE57495 (*n* = 63), GSE62452 (*n* = 130), GSE71729 (*n* = 191), GSE85916 (*n* = 80), all obtained from GEO database, and ICGC‐PACA‐UA (*n* = 269) sourced from the ICGC database. The scRNA‐seq data of our in‐house ICB clinical cohort has been deposited at Zenodo and is available at https://zenodo.org/records/10842011. Other data supporting the findings of this study are available within the article and its Supplementary Materials.
