# Supplementary material for: PLXDC1 + Tumor‐Associated Pancreatic Stellate Cells Promote Desmoplastic and Immunosuppressive Niche in Pancreatic Ductal Adenocarcinoma
Source: Adv Sci (Weinh). 2025 Mar 17;12(18):2415756. doi: 10.1002/advs.202415756 (PMC12079351; doi:10.1002/advs.202415756)

ADVANCED SCIENCE Open Access

Supporting Information

for *Adv. Sci.*, DOI 10.1002/advs.202415756

PLXDC1+ Tumor-Associated Pancreatic Stellate Cells Promote Desmoplastic and Immunosuppressive Niche in Pancreatic Ductal Adenocarcinoma

Yanhua Du, Yizhou Zhao, Judong Li, Jiaxin Wang, Shenglan You, Yao Zhang, Li Zhang, Jihong Yang, Hamid Alinejad-Rokny, Shujie Cheng, Chenghao Shao*, Duowu Zou* and Youqiong Ye**

Supplementary Figure Legend

Supplementary Figure 1. The transcriptomic and epigenetic reprograms during the activation of PSCs, related to Figure 1.

(A) Dot plots showing the average expression (dot color) and percentage of cells expressed (dot size) of the representative markers for 11 major cell types from 24 primary PDAC tumors and 11 control pancreases.

(B) Box plots showing differential percentages of PSCs between human control pancreas (n = 11) and PDAC samples (n = 24). Box middle lines, median; box limits, upper and lower quartiles; box whiskers, 1.53 the interquartile range. Statistical differences were determined using the Wilcoxon rank-sum test.

(C) Violin plot showing the distribution of activity scores for PSCs in human control pancreas and PDAC tumors, calculated as the average expression of activated PSC signature genes from Sherman et al. 2014. Statistical differences were determined using the Wilcoxon rank-sum test.

(D) Flow cytometry profiles representing screening strategies for PSCs (Lin^-/$PDPN^-$/$CD140b^+$) were depicted, with further subdivision of PSCs into $CD90^+$ and $CD36^+$ subpopulations.

(E) Bar plot showing the representative Gene Ontology (GO) enrichment for upregulated and downregulated genes defined through the comparison of differential gene expression between $CD36^+$ PSCs in adjacent normal tissues and $CD90^+$ PSCs in PDAC tumors. Hypergeometric test was performed using Benjamini-Hochberg–adjusted *P*-values.

(F) Violin plots showing the distribution of average expression score for upregulated and downregulated genes involved in PSCs activation within single-cell data derived from control pancreas and PDAC tumors. Statistical differences were determined using the Wilcoxon rank-sum test.

(G) Flowchart of integrating transcriptomic and ATAC-seq data, first merge peaks identified from different samples for unsupervised clustering into up-regulated peaks and down-regulated peaks during the activation of PSCs, then perform gene annotation

on the peaks, and combined with the expression profiles of the genes to defined peaks with co-upregulated gene expression (n=996) and peaks with co-downregulated gene expression (n=835).

(H) Heatmap of ATAC-seq tag densities were within -5/+5kb genomic regions surrounding the centers of 12,211 downregulated peaks. Samples were prepared in duplicates.

(I) The annotation of ATAC-seq peaks based on their genomic location.

(J) The IGV view showing the RNA-seq and ATAC-seq signaling profile of the loci of downregulated genes (*HES1* and *VIM* as examples). The shaded boxes represent downregulated peaks during the activation of PSCs.

(K) Transcription factor motif analysis of downregulated peaks on downregulated genes (peaks with co-downregulated gene expression, n=835) showing the significantly enriched binding elements. *P*-values were calculated using Hypergeometric distribution.

Supplementary Figure 2. Characterization of CPSCs and TPSCs in control pancreas and PDAC samples by single-cell transcriptome, related to Figure 2.

(A, B, C) Single-cell UMAP showed distribution before batch correction (A) and after batch correction using Harmony (B) or RPCA (C).

(D) UMAP plot display 7 PSCs subsets post batch correction by RPCA.

(E) Heatmap showing the Pearson correlation between PSCs subsets defined with RPCA and Harmony integration method.

(F) Bar plots showing percentages of matched PSCs subsets defined with Harmony in PSCs subsets defined with RPCA.

(G) The top 10 representative markers, transcription factors, secreted proteins, and cell membrane proteins significantly expressed by each subpopulation of PSCs are shown in the table.

(H) Feature plots showing the distribution of average expression scores for motif targeted genes and hypoxia-associated genes in each PSCs subcluster.

Supplementary Figure 3. Three distinct subcusters of TPSCs with myCAF-like, SMCs-like and inflammation-associated features, related to Figure 3.

(A) Representative gene ontology (GO) terms enriched in different CPSCs subclusters from human control pancreas samples. Hypergeometric test was performed with Benjamini-Hochberg (BH) adjusted *P* values.

(B) Heatmap showing expression of representative cytokine genes in CPSCs subclusters. Expression was normalized with z-score.

(C) Violin plots comparing the scores of *SERPINE1*$^+$ PSCs from human control pancreas and PDAC tumors in terms of vascular development (GO:1904018) and cytokine signaling (R-HSA-1280215). The scores were calculated by the average expression of each geneset. Statistical difference was determined by Wilcoxon rank-sum test.

(D) Trajectory analysis of the PSCs states by PHATE. The map showing different PSCs clusters along the differentiation trajectories.

(E, F) Kaplan-Meier survival curves for OS between patients with high and low gene signature score of *PLXDC1*$^+$ TPSCs (E) and *MYH11*$^+$ TPSCs (F) based on optimal cutoff in clinical datasets.

(G) UMAP plots display the six fibroblasts subclusters in the human control pancreas and PDAC tumors. Each dot represents one cell. Cells are color-coded by cell clusters accordingly.

(H) Dot plots showing the average expression (dot color) and percentage of cells expressed (dot size) of the representative markers for 6 fibroblasts subclusters defined in (D).

(I) Feature plots showing iCAFs and myCAFs score, which calculated by the average expression of each geneset. The signature genes for iCAFs and myCAFs were summarized in Supplementary Table 3.

(J) The Kaplan-Meier curves showing the association of overall survival (OS) time and the abundance of *LRRC15*$^+$ CAFs predicted by CIBERSORTx in TCGA cohort. The *P*-value is calculated by two-sided log-rank test.

(K) Violin plots comparing specific genesets scores between *LRRC15*$^+$ CAFs and other fibroblasts subclusters. The signature genes for PSCs-derived CAFs and ECM genes enriched in PSCs-derived CAFs were download from Helms *et al.* 2022. The scores

were calculated as the average gene expression of each geneset. Statistical difference was determined by Wilcoxon rank-sum test.

Supplementary Figure 4. Differential spatial distribution of PSCs subclusters, related to Figure 4.

(A) Spatial feature plots showing the average expression of hypoxia and Cancer_ECM genesets on HE slides. These two gene sets are summarized in Supplementary Table 3.

(B) HE images with pie charts in each spot colored by annotation showing the cellular composition in PDAC TME.

(C) Spatial feature plots showing the CNV score on HE slides (upper panel), and spatial transcriptomic spots colored by region annotation as Malignant spot (Mal), Boundary spot (Bdy) or nonmalignant spot (nMal) (lower panel).

(D) Violin plots showing the cell aboudance of three CPSCs subclusters in each TME niche.

The corresponding HE staining image was used as a background to provide a spatial location reference in A, B and D.

Supplementary Figure 5. Distinct patterns of cellular composition within each spatial spot, related to Figure 5.

(A, B) Malignant cells subclusters in different color (A) and representative markers for each subcluster (B).

(C) Feature plots showing basal-like score, classical score, proliferating score and interferon score among malignant cells. These scores are calculated based on the average expression of corresponding gene sets, which are summarized in Supplementary Table 3.

(D, E) Macrophages subclusters in different color (D) and representative markers for each subcluster (E).

(F, G) T cells subclusters in different color (F) and representative markers for each subcluster (G).

(H) Heatmap showing the pairwise Pearson correlation between the proportions of infiltrating cell clusters within each spatial spot. Here, the cellular composition within

each spatial spot was assessed using Cell2location.

(I) Estimated cell abundances (color intensity) of different cell types (color) across each spatial spot. The corresponding HE staining image was used as a background to provide a spatial location reference.

(J) Estimated cell abundances (color intensity) of T cells across each spatial spot with different shape indicating distinct spatial regions, including malignant spots (Mal), boundary spots (Bdy) or nonmalignant spots (nMal).

(K) Box plot showing the cell abundance of CD4 Treg and CD8 Tex across different spatial regions including the malignant region (Mal), the tumor boundary (Bdy) and the nonmalignant region (nMal).

(L) Estimated cell abundances (color intensity) of *PLXDC1*$^{+}$ TPSCs, CD8 Tex and basal-like Mals (color) across each spatial spot. The corresponding HE staining image was used as a background to provide a spatial location reference.

(M) Spatial feature plots showing the gene expression (color intensity) of markers (*TIGIT*, *PDCD1*, *LAG3* and *HAVCR2*) for Tex cells among each spatial spot. The corresponding HE staining image was used as a background to provide a spatial location reference.

(N) Dot plots showing the average expression (dot color) and percentage of cells expressed (dot size) of the selected markers for major cell types.

(O, P) Spatial distribution of five cell types (colors) (M) and detected probes for representative genes (colors) (N) on PDAC slices, information from ISS data. The corresponding cell segments image was used as a background to provide a spatial cell location reference.

Supplementary Figure 6. *PLXDC1*$^{+}$ TPSCs are associated with the immune-suppressive microenvironment, related to Figure 6.

(A) Heatmap showing the activity of top-ranked ligands inferred to regulate basal-like malignant cells by *PLXDC1*$^{+}$ TPSCs (left panel) and the regulatory potential of top-ranked ligands for the downstream target genes in basal-like malignant cells (right panel).

(B) Feature plots showing the average expression (color intensity) of ECM receptors

among malignant cells.

(C) Heatmap showing the activity of top-ranked ligands inferred to regulate *SPP1*$^{+}$ macrophages by *PLXDC1*$^{+}$ TPSCs (left panel) and the regulatory potential of top-ranked ligands for the downstream target genes in *SPP1*$^{+}$ macrophages (right panel).

(D) Flow cytometry profiles representing screening strategies for exhausted CD8 T cells (CD8^{+}/LAG3^{+}/PD-1^{+}) from the co-culturing of PSCs with T cells were depicted.

(E) Pearson correlation analysis of *PLXDC1*$^{+}$ TPSCs signature scores with T cell cytotoxic levels (left panel) and T cell exhaustion levels (right panel) in patients (n=8) from our in-house ICB cohort. Two-sided *P*-value for the t-distribution with n-2 degrees of freedom.

(F) Malignant cells subclusters from the ICB clinical cohort in different color (left panel), with showing the expression distribution of basal-like and classical signatures score (right panel).

(G) Macrophages subclusters from the ICB clinical cohort in different color (left panel), with showing the expression distribution of representative markers (*SPP1* and *C1QC*) (right panel).

(H, I) T cells in the ICB clinical cohort were sub-clustered and represented by different colors (F), with representative markers assigned to each subcluster (G).

(J) Box plots showing the gene expression enrichment score of cytotoxic and exhaustion signatures between responders (R) and non-responders (NR). Box middle lines, median; box limits, upper and lower quartiles; box whiskers, 1.53 the interquartile range. Statistical differences were determined using the Wilcoxon rank-sum test.

(K) Box plots showing the gene expression enrichment score of *PLXDC1*$^{+}$ PSCs signatures between responders (R) and non-responders (NR). Box middle lines, median; box limits, upper and lower quartiles; box whiskers, 1.53 the interquartile range. Statistical differences were determined using the Wilcoxon rank-sum test.

(L) The cell-cell interaction strength by Cellchat in responders (R) and non-responders (NR).

Supplementary Figure 1

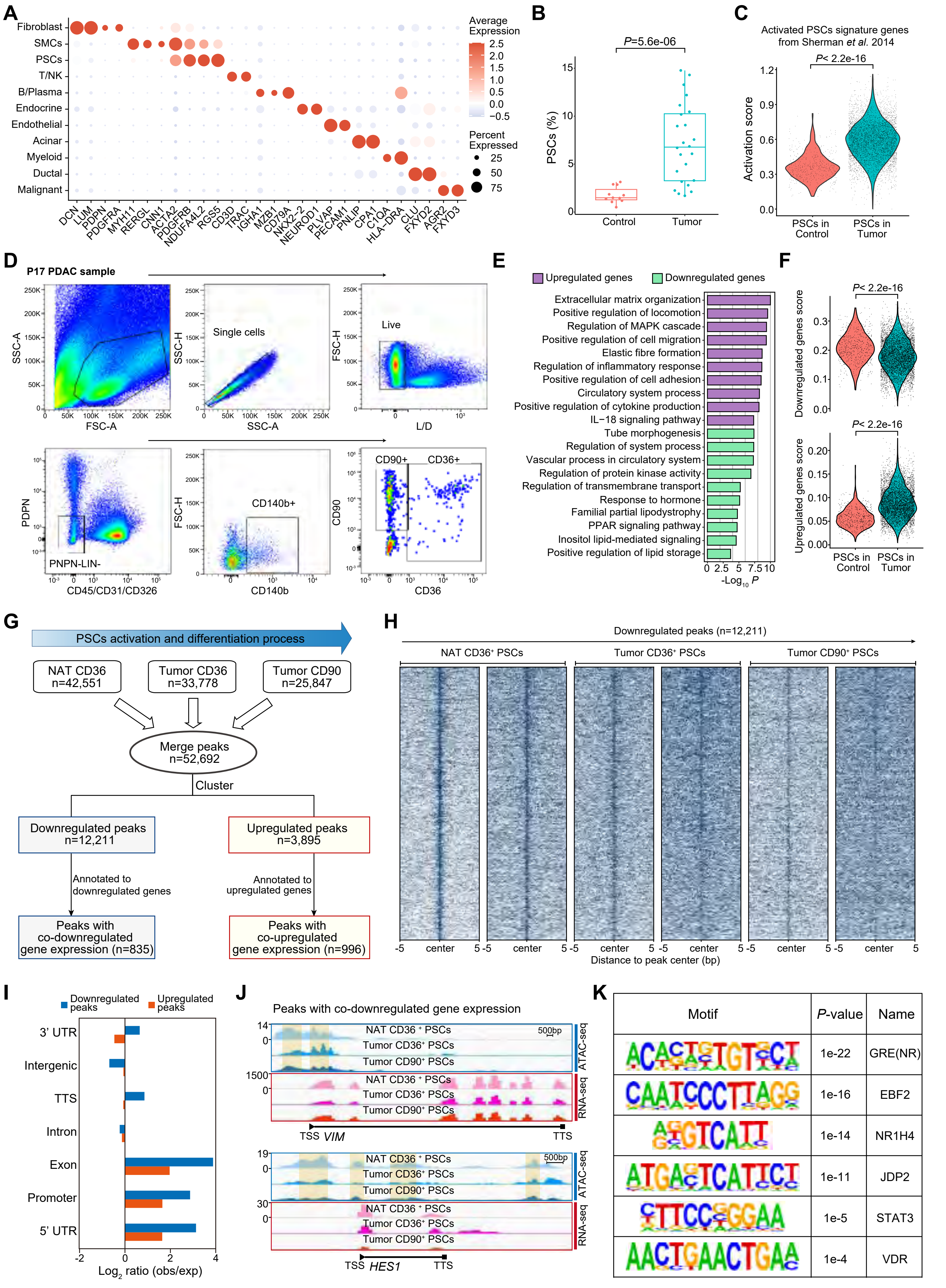

Supplementary Figure 2

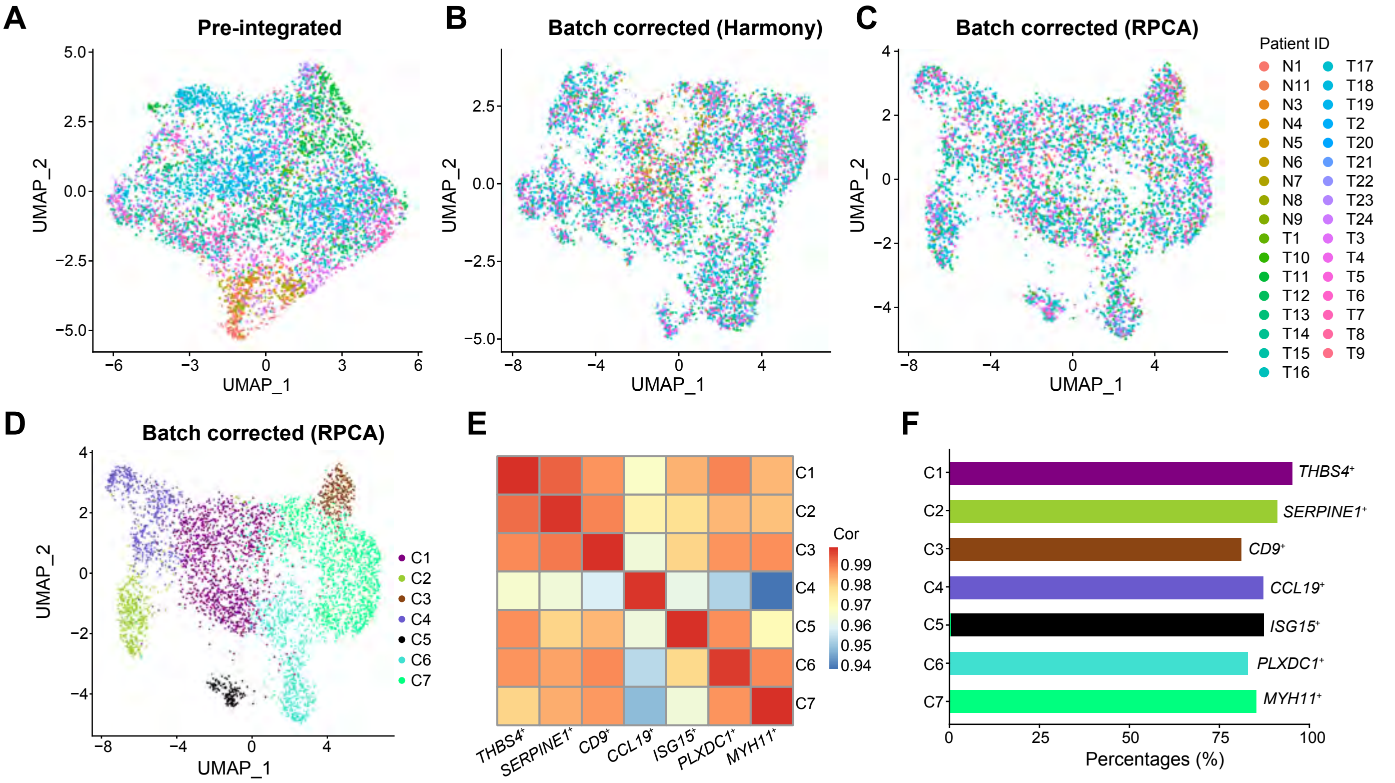

G

	Top 10	Transcription factors	Secreted	Cell membrane
THBS4$^+$ PSCs	THBS4	TSHZ2	THBS4	STEAP4
	COLEC11	TCF21	COLEC11	FAM213A
	FABP4	TBX3	IGFBP2	CD36
	STEAP4	HMOX2	GPX3	FXYD6
	TXNIP	HES1	C7	KCNK17
	FAM213A	ZBTB16	ASPN	FAM162B
	TSHZ2	ZBTB20	RARRES2	PTGER3
	CD36		C1S	HIGD1B
	IGFBP2		TIMP3	DCBLD1
	TCF21		GAS6	FAM162A
SERPINE1$^+$ PSCs	SERPINE1	FOSB	SERPINE1	EMP1
	CCL2	NR4A1	CCL2	MCL1
	CTGF	EGR1	CTGF	TNFRSF12A
	CYR61	MYC	CYR61	MYC
	FOSB	FOS	THBS1	MYADM
	THBS1	JUND	TIMP3	ADAMTS4
	EMP1	MAFF	ADAMTS4	CD36
	KLF6	ATF3	IGFBP2	TM4SF1
	MCL1	NR4A3	CD44	APOLD1
	ZFP36	ID4	NAMPT	SLC2A3
CD9$^+$ PSCs	RASD1	ID1	GPX3	CD9
	RGCC	ID3	IGFBP6	NDUFA4
	CD9	HOPX	PLAC9	HIGD1B
	ID1	JUNB	PGF	CRYAB
	GPX3	HES1	FRZB	MGST3
	FABP4	ZBTB16	CCDC3	GPRC5C
	ADIRF	CREM	PRSS1	NDUFA4L2
	RERGL		INS	SLC25A4
	ID3		SOD3	SLC38A11
	TSC22D1		SERPINI1	RCAN2

	Top 10	Transcription factors	Secreted	Cell membrane
CCL19$^+$ PSCs	CCL19	ID4	CCL19	TMEM176B
	CCL21	EGR1	CCL21	TMEM176A
	MFAP4	MYC	MFAP4	GPC3
	CCDC80	NR4A1	CCDC80	FMO2
	FGF7	FOXO3	FGF7	PCSK5
	C7	FOS	C7	TM4SF1
	CYR61	FOSB	CYR61	PROS1
	CFH	JUN	CFH	PTGER1
	APOE	PBX1	APOE	VMP1
	SOD2	CEBPB	IGFBP5	PLPP1
ISG15$^+$ PSCs	ISG15	STAT1	ISG15	IFI6
	IFIT2	IRF7	IL32	IFI27
	IFIT3	SP100	LGALS3BP	LY6E
	IFI6	SP110	TNFSF13B	BST2
	IFIT1	STAT2	APOL1	IFITM1
	IFI44L	ZBTB20	MGP	EPSTI1
	GBP1	IRF2	ASPN	VAMP5
	IFI27		TNFAIP6	RARRES3
	OAS1		SERPINI1	TNFSF13B
	GBP4		C1R	VCAM1
PLXDC1$^+$ PSCs	FN1	HIC1	FN1	PLXDC1
	COL1A1	FOXS1	COL1A1	THY1
	COL3A1	LZTS1	COL3A1	CD248
	COL1A2	MEF2C	COL1A2	KCNJ8
	COL6A3	ETS1	COL6A3	F2R
	LUM		LUM	HLA−A
	COL6A2		COL6A2	ABCC9
	COL5A2		COL5A2	AGRN
	PRSS23		PRSS23	MMP14
	PLXDC1		MMP11	S1PR3
MYH11$^+$ PSCs	S100A4	ZFHX3	WFDC1	MCAM
	MYH11	TBX2	PDGFA	MYLK
	ACTG2	MEF2C	EGFL6	PLN
	CRIP1	FOXF1	SLIT3	KCNMB1
	ACTA2	HES4	LTBP1	SDC2
	PHLDA2	HEYL	ADAMTS9	PDLIM5
	MCAM	ZMIZ1	OLFML2A	SLC38A11
	WFDC1	YBX3	FN1	NDUFA4
	TAGLN	BHLHE40	PGF	BCAM
	DSTN	MEF2D	AOC3	CSPG4

a OtherTop10 a Transcription factors a Secreted

a Cell membrane a Secreted/Cell membrance

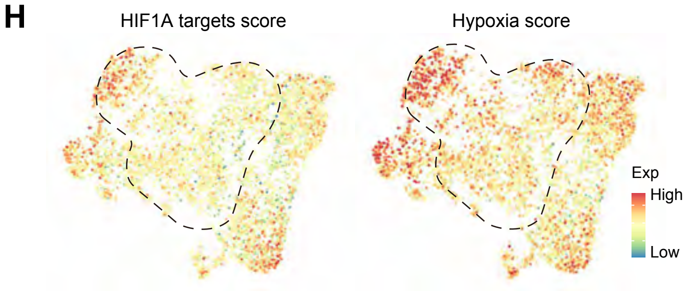

Supplementary Figure 3

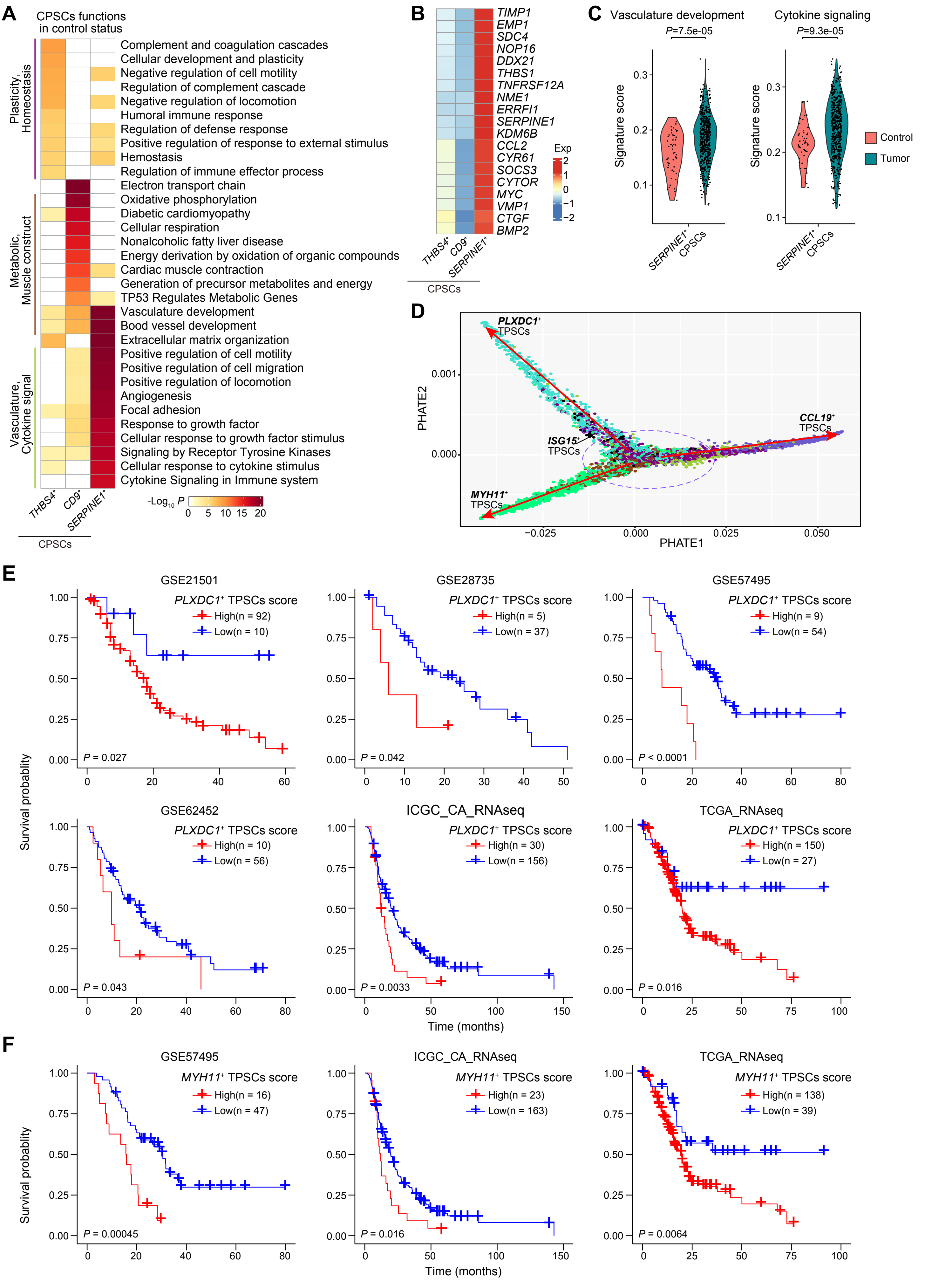

Continued Supplementary Figure 3

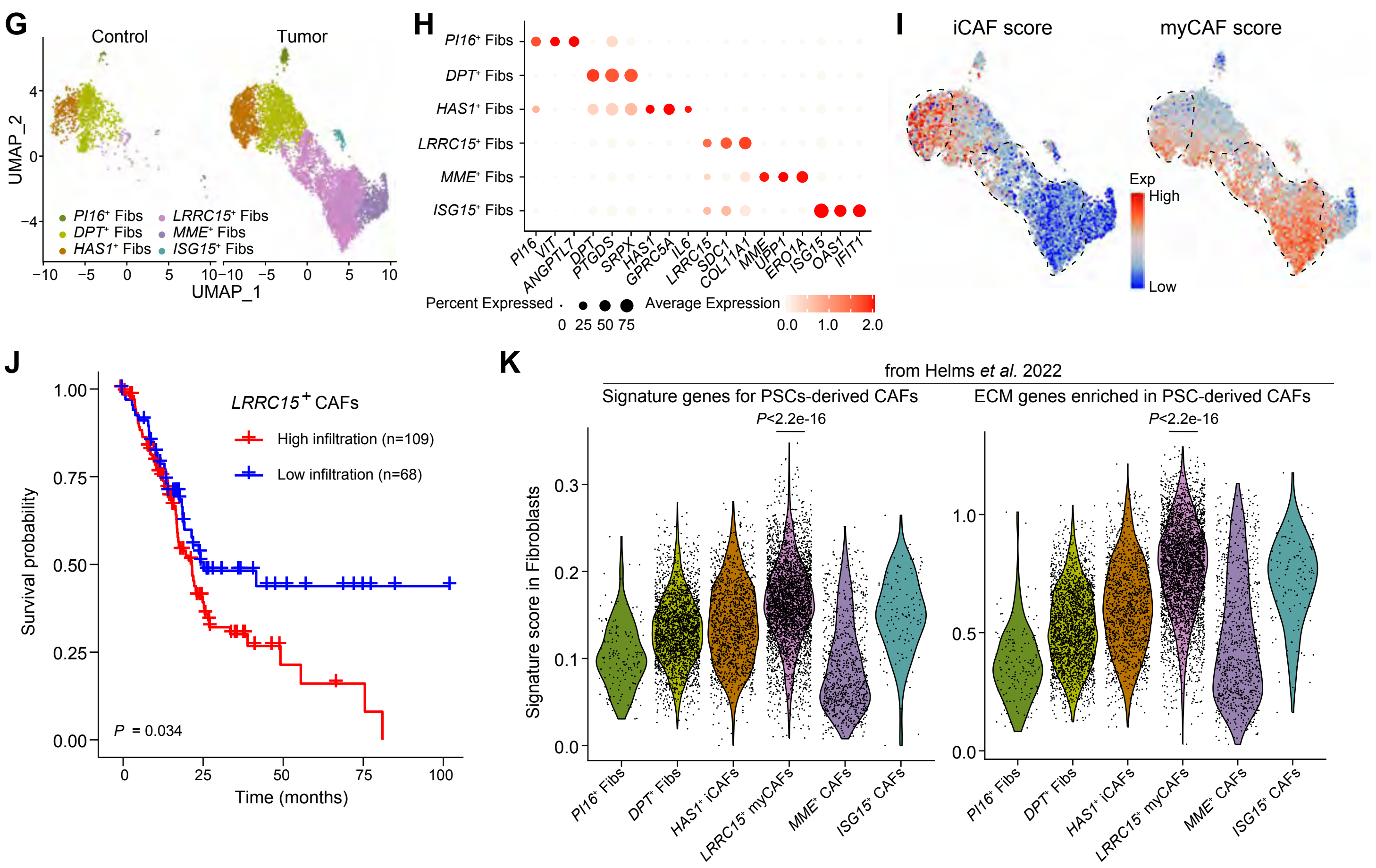

Supplementary Figure 4

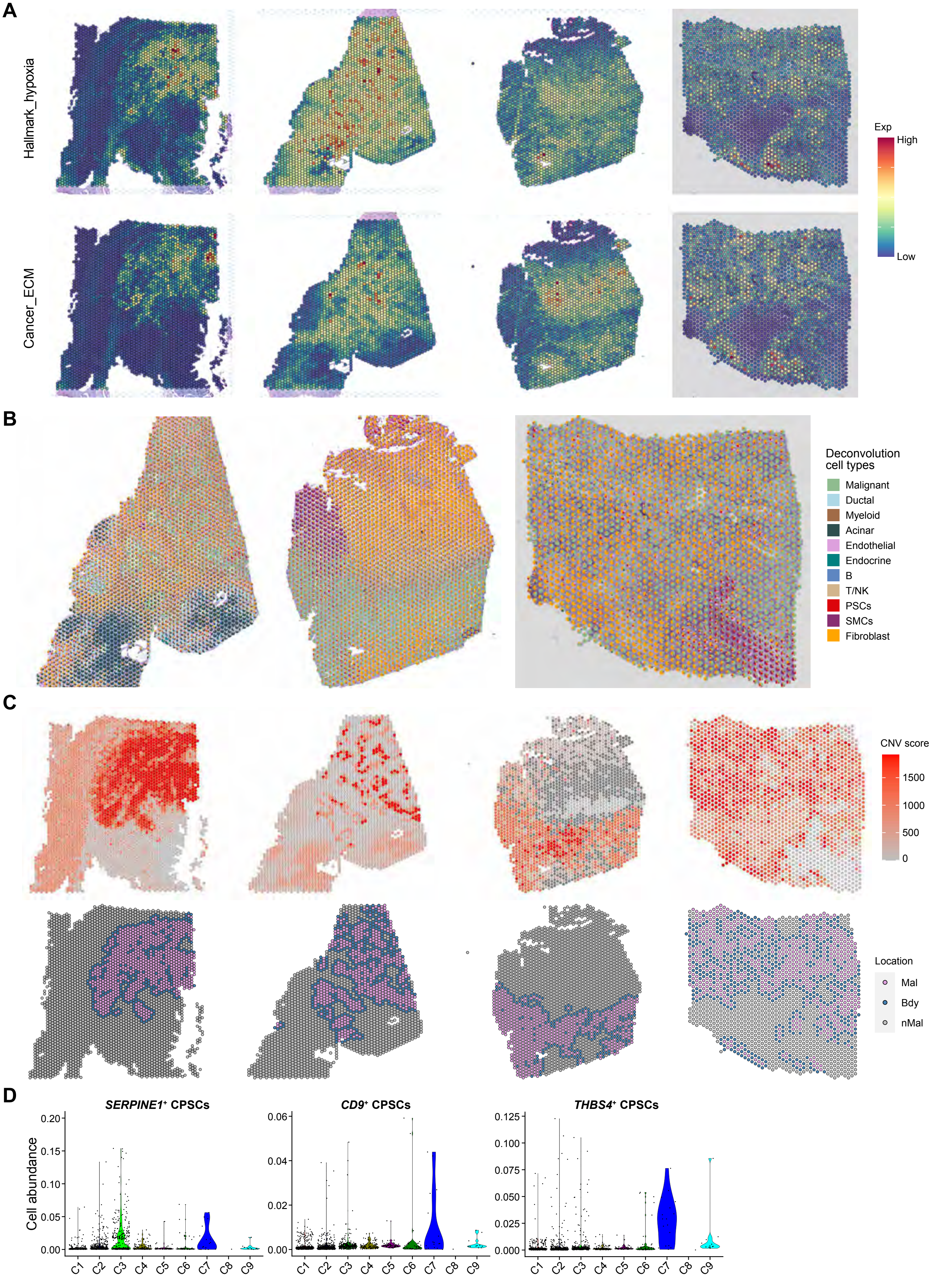

Supplementary Figure 5

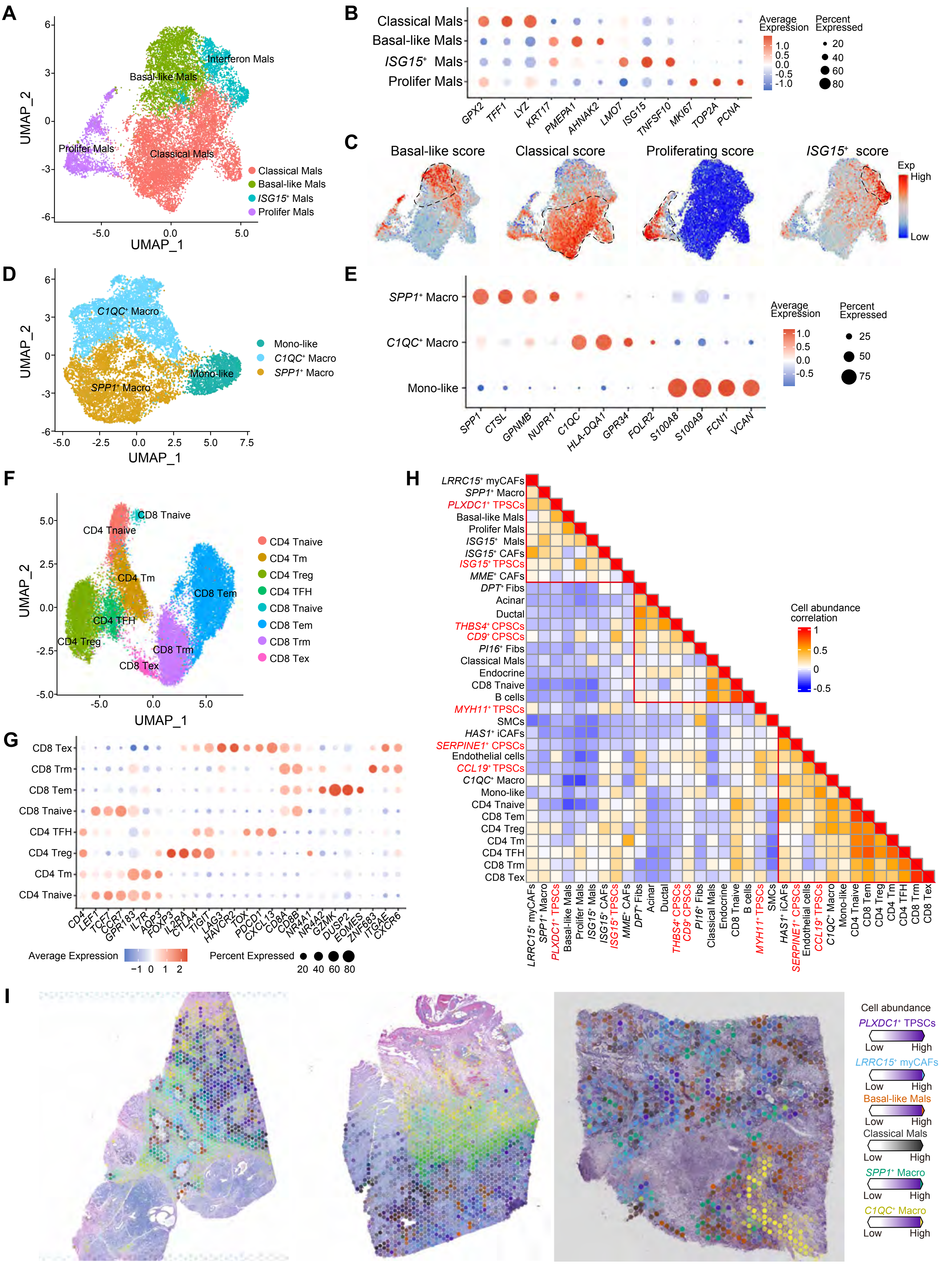

Continued Supplementary Figure 5

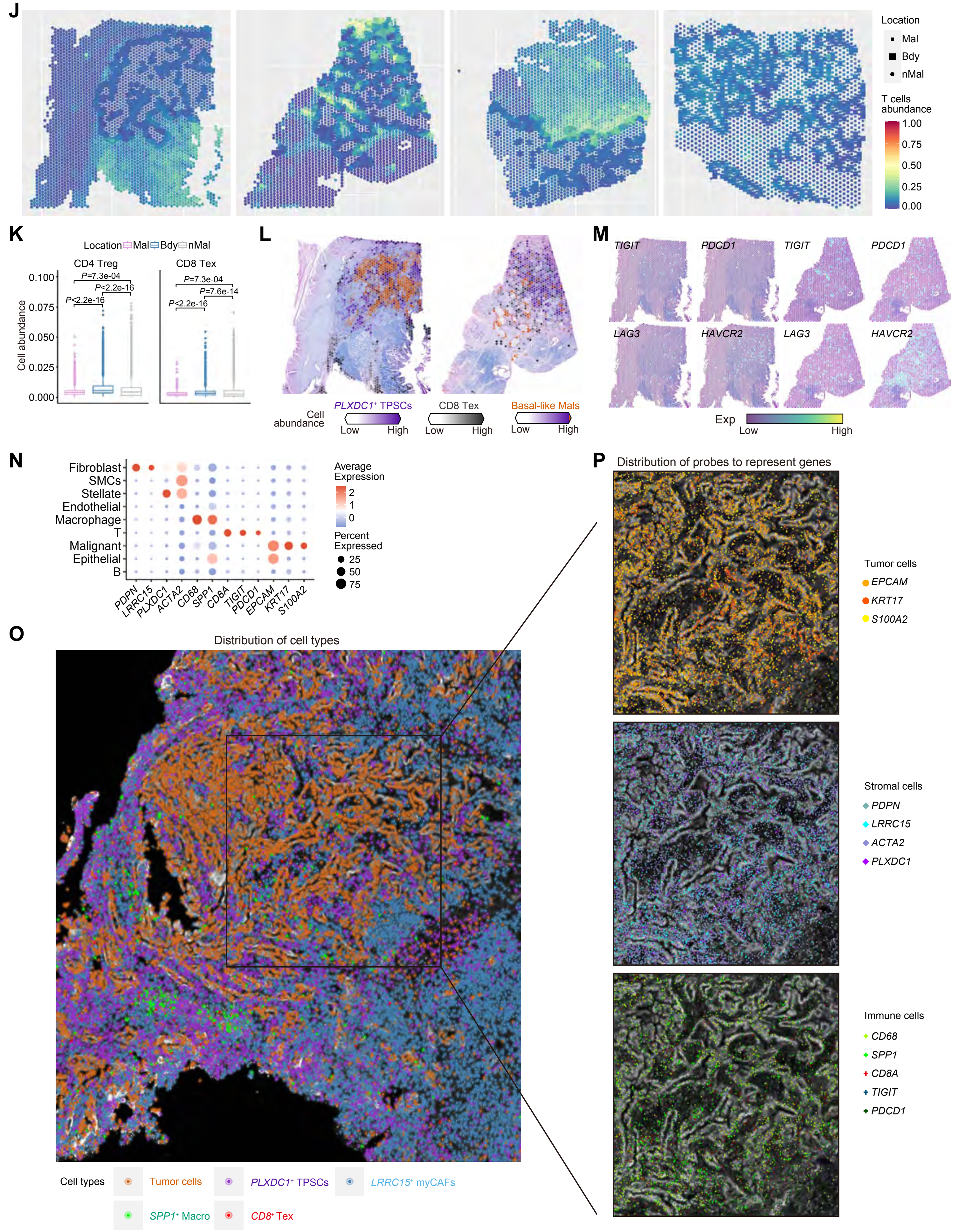

Supplementary Figure 6

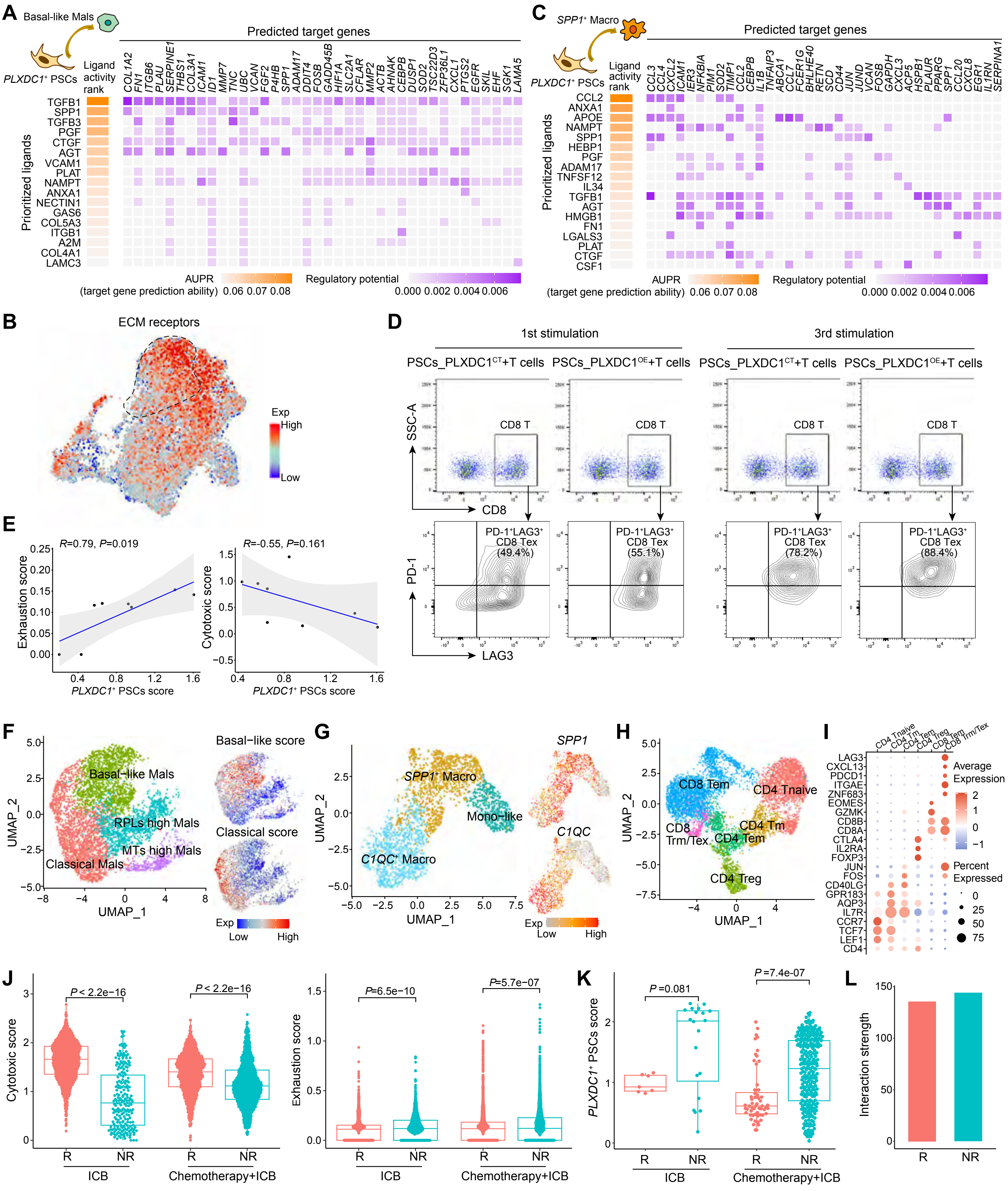

Supplement: Supplementary file 1 — Supporting Information [file ADVS-12-2415756-s002.pdf]
